# BMP suppresses WNT to integrate patterning of orthogonal body axes in adult planarians

**DOI:** 10.1371/journal.pgen.1010608

**Published:** 2023-09-20

**Authors:** Eleanor G. Clark, Christian P. Petersen

**Affiliations:** 1 Department of Molecular Biosciences, Northwestern University; Evanston Illinois, United States of America; 2 Robert Lurie Comprehensive Cancer Center, Northwestern University; Evanston, Illinois, United States of America; University of Oxford, UNITED KINGDOM

## Abstract

Adult regeneration restores patterning of orthogonal body axes after damage in a post-embryonic context. Planarians regenerate using distinct body-wide signals primarily regulating each axis dimension: anteroposterior Wnts, dorsoventral BMP, and mediolateral Wnt5 and Slit determinants. How regeneration can coordinate perpendicular tissue axes without symmetry-breaking embryonic events is not fully understood. Here, we report that the planarian dorsoventral regulator *bmp4* suppresses the posterior determinant *wnt1* to provide patterning input to the anteroposterior axis. Double-FISH identified distinct anteroposterior domains within dorsal midline muscle that express either *bmp4* or *wnt1*. Homeostatic inhibition *bmp4* and *smad1* expanded the *wnt1* expression anteriorly, while elevation of BMP signaling through *nog1;nog2* RNAi reduced the *wnt1* expression domain and elevated *bmp4* expression. Homeostatic BMP signal perturbation broadly affected anteroposterior identity as measured by expression of posterior Wnt pathway factors, and caused mislocalization of AP-regionalized pharynx progenitors, without strongly affecting expression domains of anterior regulators. Additionally, *wnt1* inhibition elevated *bmp4* expression in the tip of the tail. Therefore, dorsal BMP signals and posterior *wnt1* mutually antagonize for patterning the tail. Furthermore, homeostatic *bmp4* RNAi caused medial expansion of the lateral determinant *wnt5* and reduced expression of the medial regulator *slit*. By contrast, *nog1;nog2* RNAi restricted *wnt5* expression. Double RNAi of *bmp4* and *wnt5* resulted in lateral ectopic eye phenotypes, suggesting *bmp4* acts upstream of *wnt5* to pattern the mediolateral axis. These results indicate *bmp4* controls dorsoventral information and also, through suppression of Wnt signals, influences anteroposterior and mediolateral identity. Based on related functions across vertebrates and Cnidarians, Wnt and BMP cross-regulation could form an ancient mechanism for coordinating orthogonal axis patterning.

## Author summary

Systems that coordinate long-range communication across axes are likely critical for enabling tissue restoration in regenerative animals. While individual axis pathways have been identified, there is not yet a complete understanding of how signaling allows repatterning across 3-dimensions. Here, we report an unanticipated linkage between anteroposterior, dorsoventral, and mediolateral systems in planarians through BMP signaling. We find that dorsally expressed BMP restricts posterior and lateral identity by suppressing distinct Wnt signals in adult planarians. These results reveal a potentially ancient role for separate axis systems to interact to generate coordinated growth in adult animals.

## Introduction

Most animal forms are organized along orthogonal body axes. Bilaterian body plans typically involve head-to-tail (anteroposterior, AP), back-to-belly (dorsoventral, DV), and midline-to-lateral (mediolateral, ML) dimensions produced with high fidelity. Signals defining each body axis can function largely independently, and many animals use canonical Wnt signaling to regulate the AP axis and BMP signaling to regulate the DV axis [1–3]. Definitive body axes emerge through embryogenesis from initial asymmetries generated either by maternal cues such as oocyte polarity [4] or symmetry-breaking events such as sperm entry, cavitation, or gastrulation [3,5]. However, the ability of some species to undergo whole-body regeneration as adults suggests embryogenesis can be unnecessary to maintain and restore axis orthogonality. In planarians, acoels, and Cnidarians, patterning along individual tissue dimensions has been attributed to distinct spatial signaling pathways [6–13]. Because such organisms can re-establish body forms for many successive generations asexually, they could in principle inherit AP and DV asymmetries from each axis dimension separately. Alternatively, perpendicular axis systems might instead interact to enable coordinated growth.

The freshwater planarian *Schmidtea mediterranea* is a model for studying the principles of adult axis patterning due to its ability to regenerate nearly any surgically removed tissue and undergo perpetual homeostasis in the absence of injury. These abilities are supported by adult pluripotent stem cells termed neoblasts that differentiate into any of the approximately 150 cell types comprising the adult animal and assemble into functional and appropriately positioned and scaled tissues [14,15]. Neoblasts undergo regionalized specialization to form subsets of progenitors fated for tissues located at particular axial locations such as the eyes, pharynx, and dorsal-versus-ventral epidermal cells [16–18]. Neoblasts also hone to particular regions through their migratory ability [19–22] in order to form organs at particular locations in the body [23–25]. Therefore, spatial information is critical for controlling progenitor function in order to maintain and regenerate the planarian body plan.

Spatial organization in regeneration and homeostasis is provided by a specialized set of signaling factors termed position control genes (PCGs) that are expressed regionally from body-wall muscle [26]. Following amputations that truncate the body axis, PCG expression domains shift to provide tissue identity information, allowing for restoration of missing body regions [27,28]. The planarian AP axis is controlled by canonical β-catenin signaling involving the use of posteriorly expressed Wnts and their signaling outputs, and anteriorly expressed Wnt-inhibitors [7,9,27–30]. The Wnt ligand *wnt1* and secreted Wnt inhibitor *notum* are expressed at the posterior and anterior poles, respectively, where they organize tail and head patterning [7,9,27–29,31]. By contrast, the DV axis is established by BMP signaling. *bmp4* is expressed in a mediolateral gradient within dorsal muscle and acts to promote dorsal fates while repressing ventral fates, through feedback inhibition of a ventrally and laterally expressed *admp* homolog [6,10,32–35]. Fates along the ML axis are determined through the reciprocal antagonism of medial *slit* and the laterally expressed non-canonical Wnt ligand *wnt5* [28,36]. *slit* inhibition results in a collapse of lateral tissues, such as eyes, onto the midline, while *wnt5* inhibition causes opposite defects in which ectopic tissue, such as eyes, form laterally [28]. Inhibition of PCG factors results in mis-patterning phenotypes both in amputated animals regenerating a new blastema and also in uninjured animals using neoblasts to maintain their bodies through homeostasis [9,10,24]. Therefore, canonical Wnt, BMP, and Slit/Wnt5 signals constitutively control axis identities across three spatial dimensions. Cross-regulation among these factors has been documented previously. *bmp4*, *wnt5*, and *slit* all regulate the position of *notum*+ anterior pole progenitors to target to the DV and ML midpoint as an early step in anterior regeneration [37]. In addition, *bmp4* also controls mediolateral eye placement, lateral tissue identity, regeneration of midline tissue after transverse amputation, and blastema outgrowth after longitudinal amputation [10] and regulates the epidermal injury-induced gene *equinox* to control head outgrowth, including the formation of the *notum+* anterior pole, in transverse regeneration [38]. By contrast, *beta-catenin-1(RNAi)* animals regenerate ectopic heads that appear to have normal DV polarization, yet these outgrowths mainly occur at the lateral edge, corresponding to the surface DV midpoint [9]. These observations suggest a potentially complex set of interactions among the determinants of each individual body axis that still awaits full elucidation.

## Results

**Fig 1:**
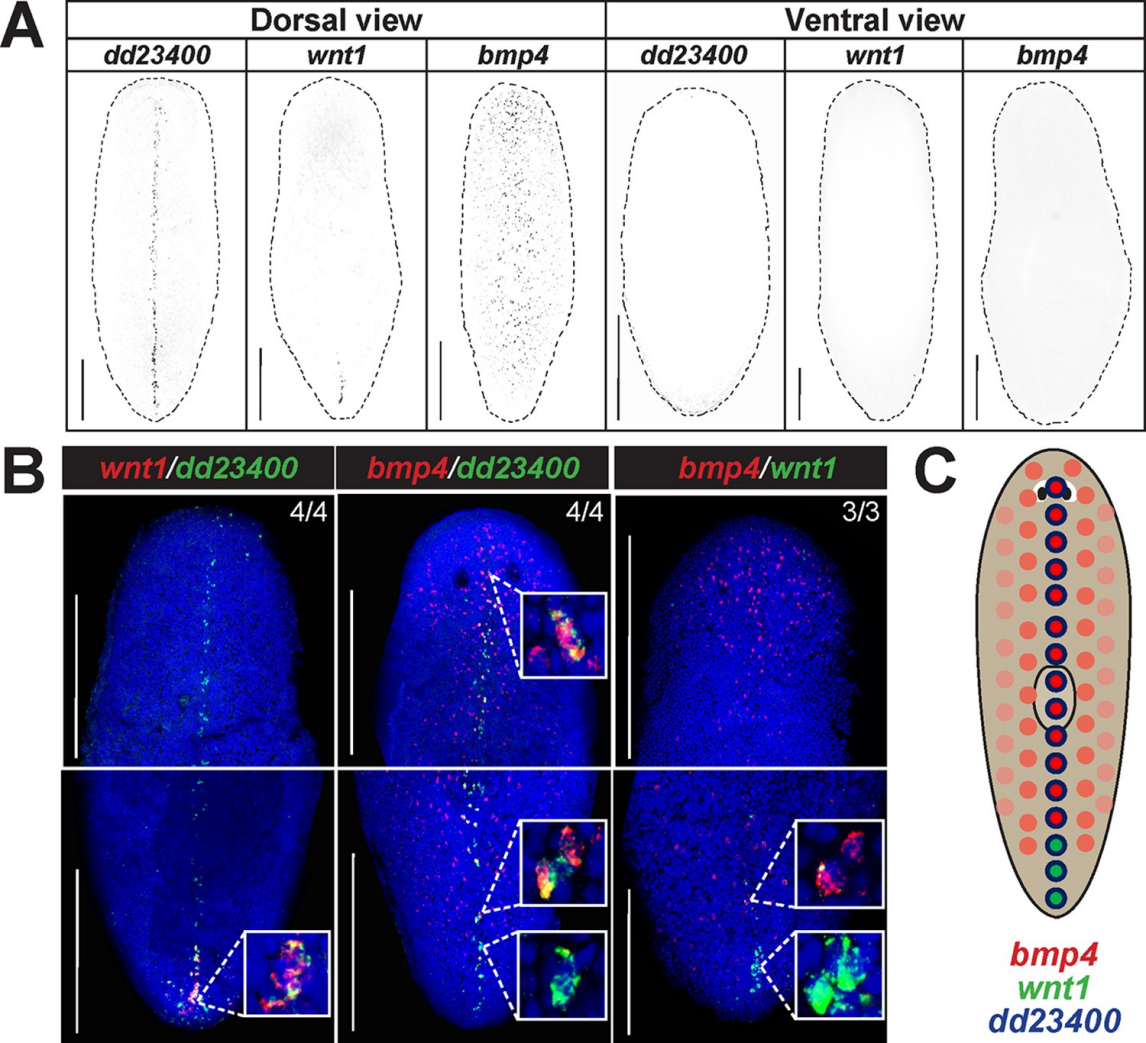
*bmp4* and *wnt1* co-express with *dd23400*+ dorsal midline cells in a regionally distinct manner. **(A)** Fluorescent *in situ* hybridization (FISH) detecting *dd23400* on the dorsal midline, *wnt1* on the dorsal posterior midline, and *bmp4* in a dorsal midline-centered gradient with reduced expression in the far posterior. Dotted line indicates animal outline. Scale bars represent 150 μm with dorsal or ventral views indicated. **(B)** Double FISH detecting co-expression of *wnt1*, *dd23400*, and *bmp4*. Left: 88.9% of the cells that were *wnt1+* were also *dd23400+* (64/72 cells from 4 animals). Middle: 55.4% of *dd23400+* cells also expressed *bmp4* (169/305 cells from 4 animals). Right: Only 12.1% of cells expressing *wnt1+* also co-expressed *bmp4* (4/33 cells from 3 animals, top inset). The *wnt1+* cells that expressed *bmp4* were consistently the anterior-most cells of the *wnt1* domain. By contrast, 87.9% (29/33 *wnt1+* cells from 3 animals, bottom inset), all located at the posterior tip, did not co-express *bmp4*. **(C)** Schematic illustrating separation of *bmp4* and *wnt1* domains on the dorsal midline. Along *dd23400+* dorsal midline muscle cells, *wnt1* and *bmp4* expression defines largely nonoverlapping AP domains within posterior. Within the *wnt1+* domain of the representative image, *wnt1+* cells in the anterior co-expressed *bmp4* whereas cells in the posterior of the domain lacked *bmp4* co-expression. Scale bars represent 150 μm.

We sought to uncover possible relationships between key determinants of orthogonal body axes in planarians. Through fluorescence *in situ* hybridization (FISH), we examined the expression of *bmp4* and observed that in addition to the prominent dorsal-versus-ventral expression pattern with highest expression on the dorsal midline [6,10,39], *bmp4* expression was stronger in the anterior versus posterior of the animal and reduced at the posterior tip [10] (Fig 1A). Furthermore, we noted that expression of *wnt1*, a master regulator of posterior identity, is selective to the dorsal and not ventral side of animals in the posterior tail approximately where *bmp4* expression is lower [9,28] (Fig 1A). *wnt1* is known to be co-expressed in a posterior subset of muscle cells specific to the dorsal midline marked by expression of *dd23400* [40]. Quantification of FISH intensity provided further support for these qualitative observations, and we compared replicate conditions by normalizing total intensity and position across either ML or AP axes (S1A–S1F Fig). *bmp4* signal was measured to be in a graded pattern along the mediolateral axis dorsally, with highest expression at the midline, while *dd23400* was expressed only in dorsal midline cells (S1A and S1D Fig). *wnt1* signal had its maximum along the dorsal midline at the same location as *dd23400* but only in the posterior (S1B and S1E Fig). Finally, comparing AP expression domains of *wnt1* and *bmp4* on the dorsal side, the *wnt1*-high regions also had relatively lower *bmp4* expression and vice-versa (S1C and S1F Fig). To further validate these models of expression behavior, we performed double FISH to examine the features of co-expression of dorsal posterior *wnt1*, dorsal *bmp4*, and dorsal midline *dd23400* (Fig 1B). *dd23400* was co-expressed in the majority (88.9%) of *wnt1*+ cells (64/72 cells from 4 animals), similar to results reported previously [40]. Additionally, *dd23400* and *bmp4* were also co-expressed, as *bmp4* expression was detected in 55.4% of *dd23400*+ cells (169/305 cells from 4 animals) on the dorsal midline. By contrast, *bmp4* was only co-expressed in 12.1% of *wnt1*+ cells (4/33 cells from 3 animals), with double-positive cells only present at the most anterior of the *wnt1* domain. By contrast, the remaining *wnt1+* cells scored (29/33 from 3 animals) were more posterior and did not co-express *bmp4*. These results suggest a model in which *dd23400+* cells are partitioned into an anterior population co-expressing *bmp4* and a population in the far posterior co-expressing *wnt1*, with a limited overlap (Fig 1C).

**Fig 2:**
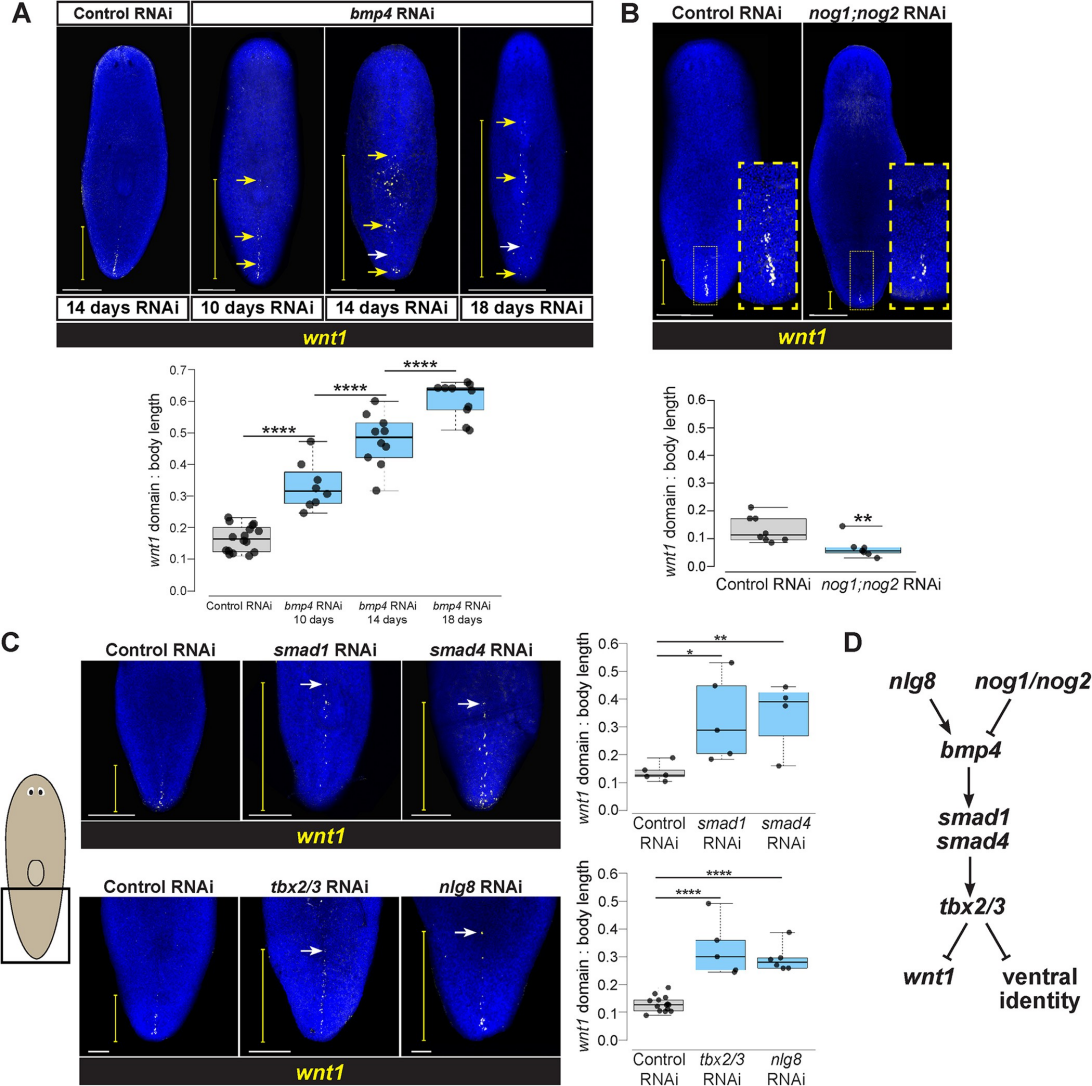
A BMP signaling pathway involved in DV identity restricts *wnt1* expression to the posterior. **(A)** FISH detecting *wnt1* expression in animals following 14 days of homeostatic control RNAi and 10, 14, or 18 days *bmp4* RNAi. Inhibition of *bmp4* expanded *wnt1* expression toward the anterior. Increased time of *bmp4* inhibition results in further anterior expression of *wnt1*. The *wnt1* domain expanded anteriorly and eventually had reduced expression in the posterior tip (yellow arrows show ectopic *wnt1+* cells, white arrows show absence of *wnt1+* cells relative to controls). **(B)** *wnt1* expression domain size was reduced in *nog1;nog2(RNAi)* animals treated with dsRNA for 18 days of homeostasis (yellow boxes, magnified insets). **(C)** Top: FISH of *wnt1* following 14 days of control, *smad1*, or *smad4* RNAi. Bottom: FISH detecting *wnt1* after 18 days of control, *tbx2/3*, or *nlg8* RNAi. Inhibition of these BMP signaling components resulted in anterior expansion of *wnt1* expression. **(A-C)** White scale bars represent 150 μm, and yellow brackets indicate *wnt1* AP expression domain size. Box plots showing the distance from the posterior tip of the animal to the most anterior *wnt1* expression relative to body length after indicated treatments. N ≥ 4 animals. Box plots show median values (middle bars) and first-to-third interquartile ranges (boxes); whiskers indicate 1.5× the interquartile ranges, and dots are data points from individual animals. *p<0.05, **p<0.01, ****p < 0.0001 by one-way ANOVA on ranks test (A,C) or by 2-tailed t-test (B). **(D)** Pathway model for components acting in DV determination and control of *wnt1*.

To test for possible functional relationships between *bmp4* and *wnt1*, we first used RNA interference (RNAi) to examine the consequences of *bmp4* inhibition. To circumvent the roles of *bmp4* in directing expression of the injury-induced *equinox* gene essential for blastema outgrowth [38], we examined axis relationships using homeostatic RNAi in the absence of injury to specifically reveal possible interactions between axis patterning factors. After *bmp4* RNAi, the *wnt1* expression domain expanded dramatically anteriorly, but retained dorsally restricted specificity (Fig 2A). This domain increase occurred by 10 days of *bmp4* RNAi and longer *bmp4* RNAi treatments of 14 and 18 days resulted in *wnt1+* cells progressing further toward the anterior of the animal. Numbers of *wnt1+* cells increased in these conditions, indicating that the expanded range of *wnt1* expression domain after *bmp4* RNAi is likely due to control of expression territory and cell abundance rather than redistribution of a set number of *wnt1+* cells (S2A Fig). The altered expression of *wnt1* in *bmp4(RNAi)* animals was more sporadic and patchier compared to the normal *wnt1* domain in control animals. Consistently, longer *bmp4* RNAi treatments also resulted in a decrease to *wnt1* expression in the posterior, suggesting both an expansion and shift of the *wnt1* expression domain. In addition, *wnt1* expression expanded laterally in *bmp4(RNAi)* animals (S2B Fig), indicative of a dual role for *bmp4* in controlling both the AP and ML domain size for posterior *wnt1* expression.

To gain insights into whether BMP signaling acts permissively or instructively in regulation of *wnt1*, we inhibited Noggin homologs *nog1* and *nog2* known to negatively regulate *bmp4* in planarian DV determination [33]. Homeostatic inhibition of *nog1* and *nog2* for 18 days decreased the *wnt1* expression domain (Fig 2B), which corresponded to a decline in *wnt1+* cell abundance (S2C Fig). Therefore, BMP signaling likely plays an instructive role in limiting posterior *wnt1* identity rather than only a permissive role.

We next examined whether this role of *bmp4* in controlling a regulator of AP identity occurred via a canonical BMP pathway signaling through Smad1 and Smad4 effectors. Planarian *smad1* and *smad4* are known to mediate dorsoventral identity along with *bmp4* [6,10]. Following a 14-day inhibition, both *smad1(RNAi)* and *smad4(RNAi)* animals had significantly anterior expansion of *wnt1* expression, phenocopying the effects of *bmp4* RNAi on *wnt1* (Fig 2C, top). Given these results, we next investigated potential regulation of *wnt1* by other factors known to act with BMP signaling to control dorsoventral identity. We examined the effects of inhibiting *tbx2/3*, a transcription factor shown to act downstream of *bmp4* for control of DV identity in *Dugesia japonica* [41]. Following 18 days of *tbx2/3* RNAi, *wnt1* expression was likewise significantly expanded toward the anterior (Fig 2C, bottom). Similarly, we investigated *nlg8*, a noggin-like gene that facilitates BMP signal activation, is expressed dorsally, and whose inhibition phenocopies the ventralization phenotypes observed after *bmp4* RNAi [33]. Homeostatic RNAi of *nlg8* for 18 days caused expansion of the *wnt1* domain (Fig 2C, bottom). While *bmp4* RNAi caused a marked increase in numbers of *wnt1+* cells and *nog1;nog2* RNAi caused a decrease, other gene inhibitions under these conditions (*smad1*, *smad4*, *tbx2/3*, *nlg8*) only affected the domain size and did not cause a statistically significant increase to absolute cell numbers (S2D and S2E Fig). We suggest it is likely this distinction from the *bmp4(RNAi)* phenotype could be due to differences in expressivity, pleiotropy, or timing of RNAi effects from inhibition of other BMP pathway components in this experiment. However, we cannot rule out the possibility of the involvement of alternative pathways or other TGFbeta-related inputs controlling this phenomenon. Taken together, these experiments provide support that a *bmp4* signaling pathway closely linked to dorsoventral identity determination acts to restrict the expression domain of the posterior determinant *wnt1* (Fig 2D).

*wnt1* also undergoes dramatic expression dynamics early in regeneration. Wound sites express *wnt1* in muscle cells early after wounding, and optimal injury-induced *wnt1* expression depends on *bmp4* signals to induce expression of the novel secreted factor *equinox*, which in turn activates many injury-induced genes [1,28,38]. In addition, regenerating tail fragments undergo an extensive remodeling of pre-existing territories so that regeneration restores the overall body proportionality without restoring absolute size. In regenerating tail fragments, *wnt1* expression undergoes an initial anterior expansion along the midline by 18 hours post-amputation, followed by eventual restriction and re-establishment of a new AP axis through rescaling over several days [28]. We tested whether *bmp4* inhibition would affect these regeneration-dependent behaviors of *wnt1* expression along the posterior midline in amputated tail fragments. In these animals, *bmp4* RNAi resulted in an anterior expansion of the *wnt1* domain from the homeostatic knockdown prior to amputation and so was present in animals fixed immediately after amputation (S3 Fig). By 18 hours, control tail fragments underwent an anterior expansion of *wnt1* along the midline, while *bmp4(RNAi)* tail fragments retained an expanded *wnt1* domain. However, *bmp4(RNAi)* animals underwent apparently normal resetting of *wnt1* domains during the rescaling period by 96 hours, similar to control animals. By contrast, a prior study found that inhibition of the STRIPAK complex factor *mob4* led to anteriorly expanded *wnt1* but loss of regeneration-induced rescaling of *wnt1* territories [40]. Therefore, although *bmp4* negatively regulates *wnt1* homeostatically, it is unlikely that the reduction to the *wnt1* expression domain through regenerative rescaling occurs through control of *bmp4* under normal conditions. Furthermore, it is likely that *bmp4* and *mob4* act separately to control *wnt1* expression.

We next tested whether the *wnt1* expansion phenotype might involve regulation of expression states in existing cells or control of cell regional identity through regulation of newly produced *wnt1+* cells. To test this hypothesis, we used sublethal X-ray irradiation to deplete neoblasts and then determine whether the *wnt1* expansion phenotype after *bmp4* RNAi is stem-cell dependent. Animals were untreated or subjected to 1350 rads of irradiation then taken through 12 days of control or *bmp4* RNAi (S4 Fig). The untreated animals robustly displayed expanded *wnt1* expression after *bmp4* RNAi conditions, compared to control RNAi untreated animals. However, irradiated control or irradiated *bmp4(RNAi)* animals lacked any detectable *wnt1* expression. We interpret these results as likely indicating that neoblasts are required for both the *bmp4(RNAi) wnt1* expansion phenotype and also to sustain the *wnt1*+ domain in control animals. We note that although a shorter-term recovery with fewer dsRNA feedings (8 days and 2 feedings) after irradiation can still support expression of *wnt1* in the tail tip in normal animals [40], in order to detect the *bmp4(RNAi)* phenotype on *wnt1*, a longer irradiation recovery and dsRNA dosing period was required (12 days and 5 feedings). We cannot rule out that irradiation has some other effect to disrupt the *bmp4(RNAi) wnt1* expansion phenotype, but given the prominent effect of irradiation in planarians to selectively deplete neoblasts, these results argue that *bmp4* RNAi likely expands and shifts the *wnt1+* domain through controlling the differentiation of cells with *wnt1+* posterior identity.

**Fig 3:**
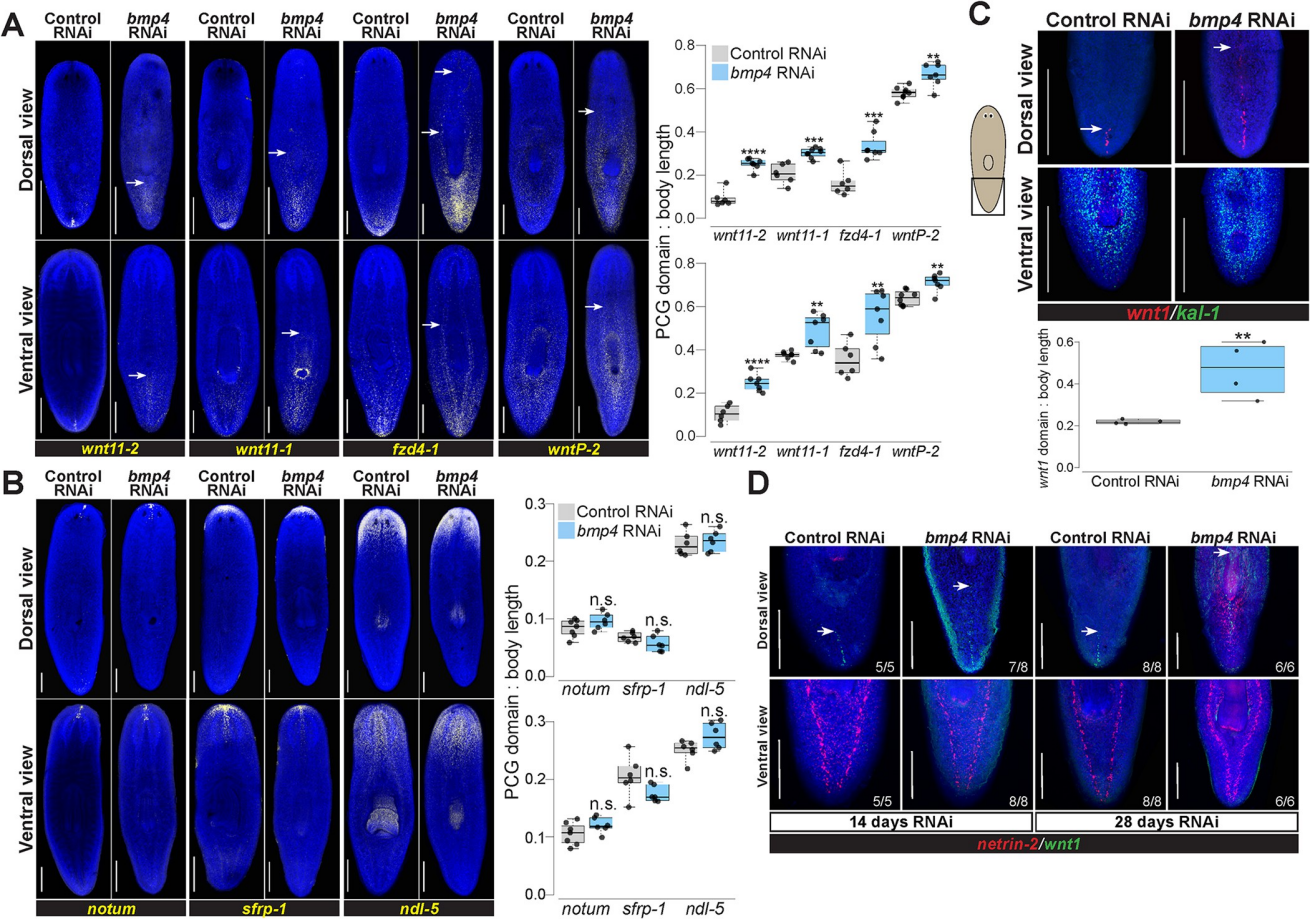
*bmp4* restricts posterior identity independent of DV control. **(A)** Animals were fixed after 28 days of homeostatic control or *bmp4* RNAi and stained by FISH as indicated for markers of AP axis identity. *bmp4* RNAi caused an anterior expansion of the posterior markers *wnt11-2*, *wnt11-1*, *fzd4-1*, and *wntP-2* on both dorsal and ventral sides. White arrows indicate the expansion of posterior markers. **(B)** FISH stained animals following 28 days of control or *bmp4* RNAi. *bmp4* inhibition did not strongly affect AP distribution of anterior markers *notum*, *sfrp-1*, or *ndl-5*. **(A-B),** Graphs show the measurement of indicated marker domains normalized by total length of animal. N ≥ 6 animals. **(C)** FISH showing *wnt1* and ventral marker *kal-1* expression comparing 14 days of control and *bmp4(RNAi)*. Dorsal view (upper) shows that inhibition of *bmp4* expanded *wnt1* expression anteriorly (arrows indicate anterior-most *wnt1+* cell) at a time prior to any dorsal expression of the ventral marker *kal-1* expression (4/4 animals). Boxplot comparing *wnt1* expression normalized by body length between control and *bmp4(RNAi)* animals. N = 4 animals. **(D)** Dorsal (upper) and ventral view of animals fixed after 14 or 28 days of control or *bmp4* RNAi and stained with *wnt1* and ventral muscle and ventral nerve cord marker *netrin-2*. *wnt1* expression expanded in *bmp4(RNAi)* animals by day 14 at a time prior to expression of *netrin-2* dorsally while netrin-2 expression was prominently dorsal by day 28. N ≥ 5 animals. **(A-C)** Box plots shows median values (middle bars) and first-to-third interquartile ranges (boxes); whiskers indicate 1.5× the interquartile ranges and dots are data points from individual animals. *p<0.05, **p<0.01, ***p < 0.001, ****p<0.0001, n.s. indicates p>0.05 by 2-tailed t-test. Scale bars, 300 μm (A-B) or 150 μm (C).

To investigate whether the role of BMP signaling was limited to *wnt1* or more broadly affected posterior identity in general, we assessed expression of other posterior markers following 28 days of *bmp4* RNAi. Posterior markers *wnt11-2*, *wnt11-1*, *fzd4-1*, and *wntP-2* are expressed in successively broader posterior domains, have roles in tail and trunk patterning [7,24,30,31,42], and are expressed in a beta-catenin-dependent manner [43–45]. Most known posterior markers such as these factors are expressed both dorsally and ventrally, unlike *wnt1*, but in regeneration their expression depends on *wnt1* [27,28]. *bmp4* inhibition expanded the domains of all four posterior markers in both their dorsal and ventral domains (Fig 3A). Therefore, BMP, a dorsal signal, broadly limits posterior identity across the dorsal and ventral axes.

By contrast, markers of far anterior identity did not appear to become restricted under these conditions of homeostatic *bmp4* RNAi. We stained *bmp4(RNAi)* animals for *notum*, *sfrp-1*, and *ndl-5* in order to assess AP identity over a range of the anterior region [7,9,28,29]. Compared to control animals, there was no significant change in these expression patterns in the AP direction on either dorsal or ventral side of the animals (Fig 3B). Quantification of *notum+* cells following *bmp4* RNAi revealed no change in absolute number or relative number of anterior pole cells (S5 Fig). We note, however, that decapitated and regenerating *bmp4(RNAi)* animals have been shown to undergo a dorsal shift to the location of their anterior pole [46], and eventually form dorsal cephalic ganglia and an extra set of dorsal eyes [10]. These transformations may impact anterior pattern expression to some degree, and we noted that the domain of *ndl-5* expression, present throughout the head of normal animals, appeared mediolaterally modified after homeostatic *bmp4* RNAi. However, our data indicate a strong role of *bmp4* homeostatically in regulation of posterior gene expression.

Because *bmp4(RNAi)* animals undergo a progressive ventralization, we considered the possibility that ventral tissue identity might indirectly influence *wnt1* expression in these animals. To ascertain possible relationships between ventralization and posteriorization phenotypes, we examined *bmp4(RNAi)* animals at an early time in their phenotypic progression after 14 days of dsRNA feeding, then simultaneously assessed both phenotypes. These animals had expanded *wnt1* but not yet dorsal expression of the ventral epidermal marker *kal-1* (Fig 3C). However, longer-term *bmp4* RNAi ultimately results in the dorsal expression of *kal-1* as the epidermis becomes ventralized during tissue turnover [16]. To provide further demonstration of the order of these events, we assessed another ventral-specific tissue by staining for *netrin-*2, which marks the ventral nerve cords and a muscle domain [47]. Similar experiments were performed with day 14 and day 28 time points of homeostatic *bmp4* RNAi, and animals were co-stained for *wnt1* and *netrin-2* to evaluate posteriorization and ventralization simultaneously (Fig 3D). Day 14 *bmp(RNAi)* animals had expansion of *wnt1* and no detectable dorsal expression of *netrin-2*, but by day 28 such animals had considerable dorsal *netrin-2* expression. Therefore, our results suggest that the anterior *wnt1* expansion after *bmp4* RNAi is unlikely a secondary consequence of tissue ventralization and instead could represent a separate use of BMP signaling for planarian AP axis patterning.

Because *bmp4* inhibition altered AP domains of posterior PCG factors, we sought to determine if inhibition of *bmp4* also affected AP determination of any differentiated tissues or AP-restricted progenitors. We compared control and *bmp4* RNAi animals to assess intestine and pharynx markers (S6A Fig). The intestine maker *porcupine* showed no change following *bmp4* RNAi. *SMU15007112*, which marks a small population of cells adjacent to the posterior end of the pharynx, did not change its position with loss of *bmp4*. In *bmp4* RNAi, the overall position of pharynx marker *dd554* staining with respect to the body axis was normal relative to body size (S6A and S6B Fig), and animals did not form an ectopic pharynx, suggesting that location of the pharynx under these conditions was unaffected by *bmp4* RNAi. However, we noticed that in control animals, *dd554* expression forms short streams of cells located radially away from the anterior portion of the pharynx, and these were absent in *bmp4(RNAi)* animals (S6A Fig). Based on single-cell RNAseq, irradiation and neoblast depletion studies, *dd554* has been suggested to mark migratory progenitor intermediates for at least some pharyngeal cell types [48,49]. Whether *bmp4-*dependent *dd554* cell streams represent a migratory progenitor state is unknown, so we examined possible effects on the pharynx progenitor marker *foxA*, which is expressed in specified neoblasts in the central region of the body surrounding the pharynx [18,50]. Homeostatic *bmp4* RNAi reduced *foxA* expression in the normal vicinity of the pharynx but strongly elevated its expression elsewhere in the body at more anterior and posterior locations. Similarly, inhibition of *bmp4* reduced expression of the pharynx marker *laminin* within the pharynx but its expression became elevated in bilateral anterior locations associated with the head. Within the cohort of animals tested in this experiment, individuals with relatively more reduction to *laminin* expression in the pharynx also had the most elevated expression in the anterior (3 of 8 animals). The mis-regulation of *dd554*, *foxA*, and *laminin* may indicate complex ways that *bmp4* regulates either specification or targeting of AP-restricted progenitors and/or differentiated cells. One possible explanation for these results is that in *bmp4(RNAi)* animals a fraction of pharynx progenitors are unable to correctly target to the pharynx and become mistargeted to aberrant locations.

We also measured effects on the AP position of eyes in homeostatic *bmp4(RNAi)* animals. *bmp4(RNAi)* animals form an extra set of medial eyes, and we noticed that long-term *bmp4* RNAi resulted in a consistent displacement of the original versus ectopic eyes in these animals. Measurements of the relative position of these eyes with respect to the anterior tip revealed that the original eyes became located too anteriorly while the ectopic eyes had exactly the same relative position as control eyes (S6C Fig). Other methods to disrupt eye patterning in planarians, for example through homeostatic *notum* RNAi, cause ectopic eyes to form at positions located correctly with respect to body landmark tissues and PCG expression domains [25]. By contrast, pre-existing eyes in such conditions can self-sustain in spite of an incorrect location, due to the flexible targeting mechanisms used by migratory eye progenitor cells [23,25]. Therefore, we interpret our results to indicate that homeostatic *bmp4* RNAi likely caused an AP tissue transformation that displaced pre-existing eyes anteriorly. Taken together, our analysis indicates *bmp4* inhibits posterior Wnts and also controls varied aspects of AP cell-type organization including pharyngeal progenitors and eye location.

**Fig 4:**
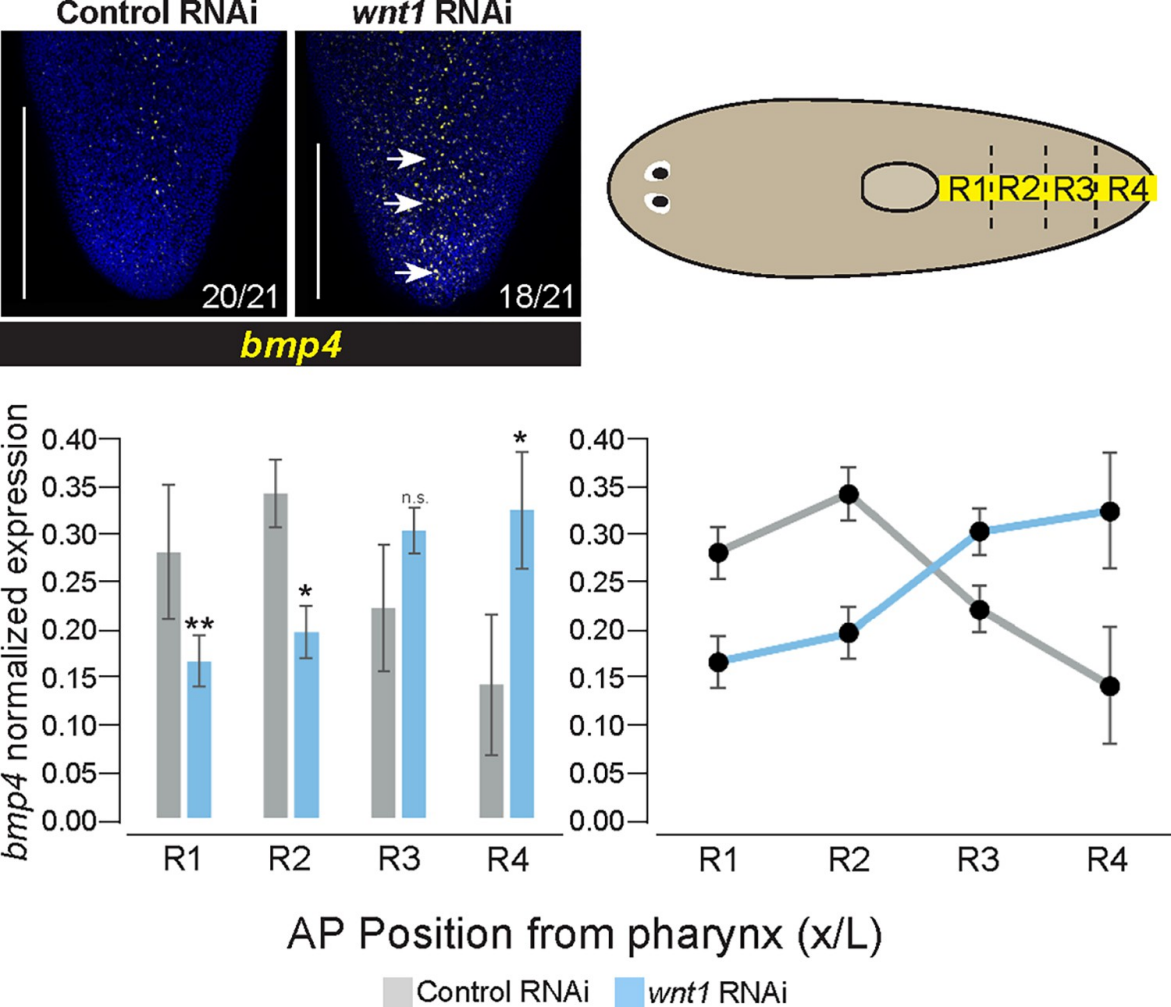
*wnt1* inhibits *bmp4* expression in the posterior. FISH staining for *bmp4* expression in the tail after 14 days of control or *wnt1* RNAi homeostatically. Dorsal views, arrows indicate observed ectopic *bmp4* expression. Number of animals scored qualitatively as normal or elevated are indicated in the panel. Scale bars are 150 μm. N = 21 animals. Upper right cartoon depicts strategy for quantification of FISH signal. FISH intensity was measured using ImageJ along the midline using a linescan approximately 1/10 the width of the animal fragment at the pharynx and measured between the tip of the tail and posterior of pharynx, across 3 biological replicates for each sample. AP positions were normalized and intensity normalized to total intensity across this range, and individual intensities were summed across 4 equal-sized regional bins. Plots show histograms (left) and line-graphs (right) of the average intensity of each bin across biological replicates, bars show standard deviations. *p<0.05, **p<0.01, n.s. indicates p>0.05 by 2-tailed t-test applied between mean binned intensities for each region measured across biological replicates. *wnt1* inhibition led to an overall increase in the relative amount of *bmp4* expression in the tip of the tail (R4).

Given that *bmp4* and *wnt1* expression was enriched in separate anterior and posterior domains, we further examined whether these genes might undergo mutual negative regulation. To test this possibility, we inhibited *wnt1* homeostatically, followed by FISH to detect expression of *bmp4*. Following 14 days of *wnt1* RNAi, tails began to retract and become bulged, as reported previously [40]. In these animals, the *bmp4* expression pattern was altered to be expressed more highly in the far posterior of the animal but retained its dorsal specificity and overall pattern elsewhere in the body (Figs 4 and S7A). Measurements of normalized FISH intensity of *bmp4* expression in the posterior demonstrate that this difference is significant. Therefore, inhibition of *wnt1* permitted *bmp4* expression in the dorsal posterior tip of the animal in a domain normally expressing *wnt1*. Together with the prior results, these experiments suggest a reciprocal antagonism between *wnt1* and *bmp4* to define each other’s expression boundaries and consequently pattern the posterior.

We also explored other possible regulation that could impact *bmp4* expression domains. In regeneration, *wnt1* controls head-versus-tail identity determination [27], but homeostatic *wnt1* knockdown has only been reported to influence tail identity [40,51]. However, to exclude the possibility that the effects of homeostatic *wnt1* inhibition on *bmp4* expression domains might be explained by head-tail polarity reversal, we stained these animals for anterior pole marker *foxD*. These *wnt1(RNAi)* conditions resulted in *bmp4* far posterior expansion and no expression of *foxD* in the posterior (S7B Fig). Therefore, the *wnt1* and *bmp4* regulation observed here is not likely due to a switch in pole identity determination. We also further explored possible influences of the anterior pole on *bmp4* domains by inhibiting *notum* homeostatically. However, *notum(RNAi)* animals did not appear to have modifications to the gradient of *bmp4* within the tail and did not alter the dorsal-specific expression of *bmp4* under these conditions (S7C Fig). We also tested whether *nog1;nog2* RNAi influenced *bmp4* expression levels in the tail. We found that *nog1;nog2(RNAi)* animals had qualitatively higher expression of *bmp4* (S7D Fig). The qualitative increase in signal intensity of *bmp4* following *nog1;nog2* RNAi suggests *bmp4* expression could be under positive feedback control from BMP signaling.

**Fig 5:**
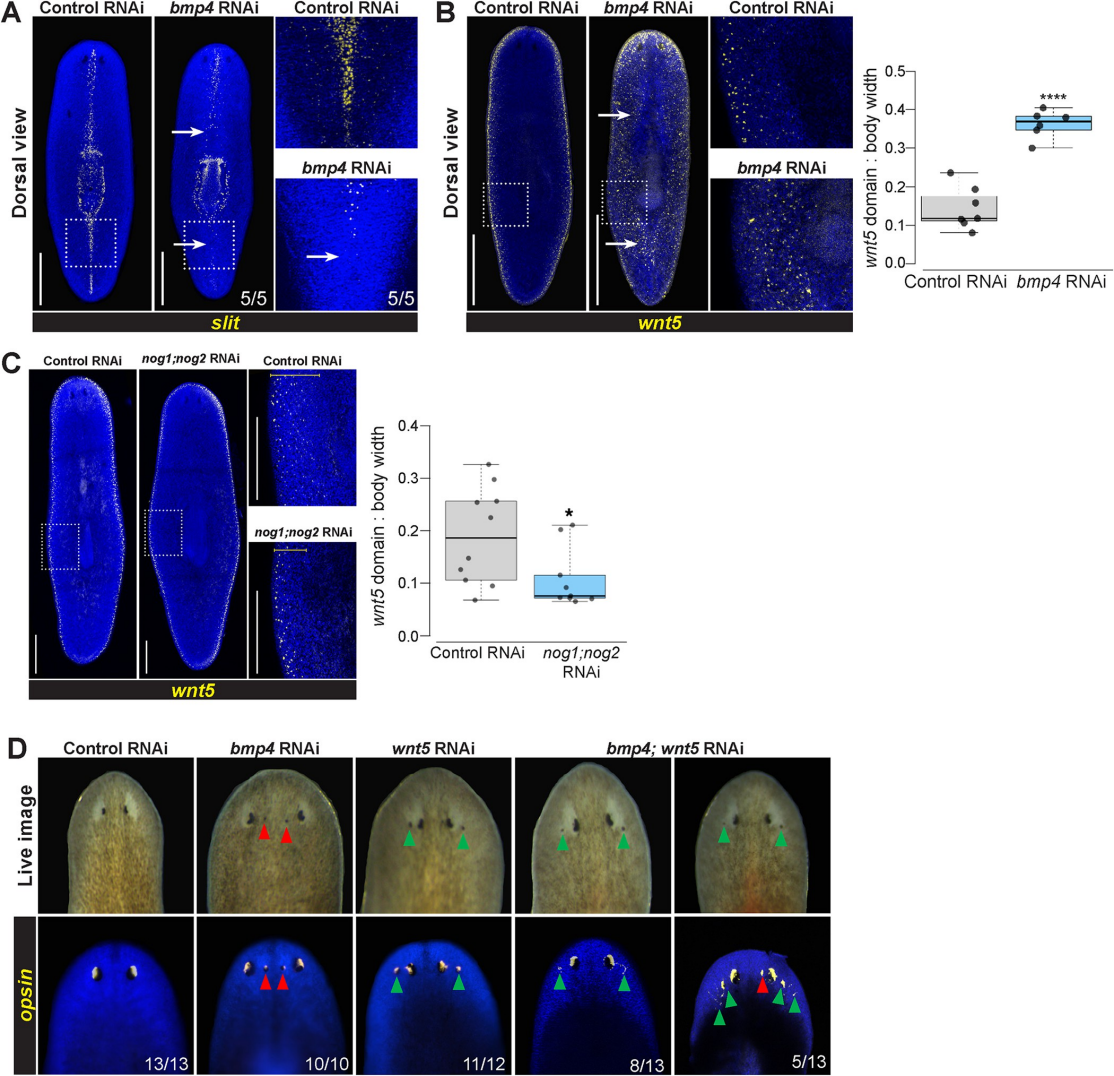
*bmp4* regulates ML patterning upstream of lateral *wnt5*. **(A-C)** FISH detecting *slit* or *wnt5* following 28 days of homeostatic control or *bmp4* or *nog1;nog2* RNAi. Right panels show enlargements of boxed regions. Scale bars represent 300 μm. **(A)** *bmp4* RNAi caused reduction of *slit* in the anterior and elimination in the posterior (arrows). **(B)** Inhibition of *bmp4* caused medial expansion of *wnt5* (arrows). **(C)** RNAi of *nog1;nog2* reduced the size of the *wnt5* lateral expression domain. **(B-C)** Graphs show *wnt5* expression domain width (dorsal side) normalized to body width. *p<0.05, ****p<0.0001 by unpaired 2-tailed t-test; N ≥ 6 animals. Box plots show median values (middle bars) and first to third interquartile ranges (boxes); whiskers indicate 1.5× the interquartile ranges and dots are data points from individual animals. **(D)** Eyes assessed by live imaging (top) or *opsin* FISH (bottom) after 21 days of homeostatic RNAi to inhibit *bmp* and/or *wnt5* and scored for lateral (green arrows) or medial ectopic eyes (red arrows). For single-gene RNAi, dsRNA for the targeted gene was mixed with an equal amount of control dsRNA so that the total amounts of each targeted dsRNA delivered were equal between single- and double-RNAi conditions. 100% of *bmp(RNAi)* animals had ectopic medial eyes and 92% *wnt5(RNAi)* animals had ectopic lateral eyes. By contrast, 100% of *bmp4;wnt5(RNAi)* animals had at least two lateral ectopic eyes, and of these 38% also formed a single medial ectopic eye (right panels) while 62% only formed ectopic lateral eyes (left panels). Co-inhibition of *wnt5* reduced the penetrance of the medial ectopic eye phenotype due to *bmp4* inhibition (p = 0.0027, 2-tailed Fisher’s exact test).

In light of the unexpected role of BMP signals in AP axis patterning, we next sought to clarify how *bmp4* participates in ML axis regulation. *bmp4* RNAi causes lateral tissue expansion and also failure to produce lateral tissue after lateral amputations [6,10]. In addition, *bmp4* RNAi causes transverse regeneration to proceed with midline indentations [6,10], likely because of *bmp4*’s role in dorsoventrally positioning the *notum+* anterior pole during head blastema outgrowth [46]. Furthermore, *bmp4(RNAi)* homeostasis animals form an extra set of eyes medially, consistent with this factor having additional roles in ML axis formation [6,10]. However, the relationship between *bmp4* and other ML axis patterning factors *slit* and *wnt5* is not fully understood. We next examined *bmp4’s* role in homeostatically maintaining midline marker expression. Following 28 days of *bmp4* RNAi, expression of the midline determinant *slit* was reduced, particularly in the posterior of the animal (Fig 5A). These results suggested that *slit* might function downstream of *bmp4* for controlling midline information. Furthermore, inhibition of *bmp4* reduced and disrupted the *dd23400* midline expression pattern and expanded its expression domain laterally (S8A Fig), suggesting BMP controls midline identity broadly. Taken together, these data suggest that *bmp4* promotes medial identity and is important for establishing the boundaries of medial territories.

We next considered how *bmp4* might interact with lateral regulatory factor *wnt5*. We first used the lateral epidermal marker *laminB* to confirm prior results that *bmp4* inhibition generated ectopic lateral tissue (S8B Fig) [32]. We then examined a possible regulatory relationship between *bmp4* and the lateral determinant *wnt5*. Following 28 days of *bmp4* RNAi, expression of *wnt5* significantly expanded to occupy distant medial territories on the dorsal side (Fig 5B), and more weakly expanded *wnt5* expression on the ventral side (S8C Fig). Furthermore, we tested whether BMP pathway activation by *nog1;nog2* RNAi might oppositely affect *wnt5* expression. Indeed, *nog1;nog2* inhibition restricted the *wnt5* expression domain (Fig 5C). Together, these results suggest that BMP signaling levels might instructively determine the location of *slit+* and *23400+* tissue at its highest levels medially and *wnt5+* identity at the lowest levels laterally.

We next tested whether *bmp4* and *wnt5* undergo reciprocal negative regulation by inhibiting *wnt5* for 21 days and staining for *bmp4*. Inhibition of *wnt5* did not cause an apparent increase or decrease of the dorsal *bmp4* gradient under these conditions (S9 Fig). In some *wnt5(RNAi)* animals, sporadic *bmp4+* cells were detected ventrally, although these lacked a clear spatial enrichment and were small in number. Because the prominent dorsal mediolateral expression gradient of *bmp4* was normal in these animals, these results suggested that *wnt5* may not regulate mediolateral identity through control of *bmp4* expression dorsally, but it may have a subtle or indirect role on dorsoventral determination. Together, these experiments indicate *bmp4* strongly antagonizes *wnt5* expression, promotes medial identity, and suppresses lateral identity.

To determine whether *bmp4* functionally controls ML identity in part through regulation of *wnt5*, we conducted epistasis tests using eye placement as a readout. *bmp4* RNAi causes the formation of ectopic medial eyes, whereas *wnt5* RNAi produces an opposite defect of the formation of ectopic lateral eyes [6,7,10]. To test for interactions between *bmp4* and *wnt5*, we homeostatically inhibited these genes individually or together for 21 days, examined animals visually, and stained them with an *opsin* riboprobe to label photoreceptor neurons (Fig 5D). Control animals had no ectopic eyes (13/13 animals), while 100% of *bmp4(RNAi)* animals (10/10 animals) had ectopic medial eyes, and 92% of *wnt5(RNAi)* animals (11/12 animals) had lateral eyes. In *bmp4*;*wnt5(RNAi)* animals, however, 100% of animals (13/13 animals, left) had at least two lateral ectopic eyes. Of these animals, 38% (5/13 animals, right) also had a single medial ectopic eye, while no animals displayed the *bmp4(RNAi)* phenotype of only medial ectopic eyes. Therefore, *wnt5* likely does not operate exclusively upstream of *bmp4*, because double-RNAi animals all displayed the *wnt5(RNAi)* lateral ectopic eye phenotype. Additionally, the two factors likely do not operate fully independently because *wnt5* co-inhibition reduced the penetrance of the *bmp4(RNAi)* medial ectopic eye phenotype (p = 0.0027, 2-tailed Fisher’s exact test). To ensure these results were not due to an inefficient *bmp4* knockdown, we used quantitative real-time PCR to evaluate normalized expression of *bmp4* and *wnt5* across each condition (S10 Fig). This experiment demonstrated that *bmp4* was significantly knocked down after treatment with both *bmp4* dsRNA and treatment with *bmp4*;*wnt5* dsRNAs. Similarly, *wnt5* was significantly knocked down in animals treated with *wnt5* dsRNA and also treated with *bmp4*;*wnt5* dsRNA (S10 Fig). We cannot rule out an influence of differential depletion of the *wnt5* and *bmp4* proteins in this experiment as contributing to the outcome, though enough protein knockdown occurred in the single-RNAi conditions to generate nearly 100% penetrant patterning phenotypes. Taken together with the findings that *bmp4* RNAi causes expansion of *wnt5* expression, we interpret these results to indicate that *bmp4* can act upstream to limit *wnt5* in order to regulate ML identity.

Given the ability of *bmp4* signaling to regulate *wnt5* expression domains and also to promote *slit* expression, we tested whether *bmp4’s* function on midline determinant *slit* might depend on negative regulation of *wnt5*. To test this hypothesis, we performed single and double knockdown experiments between *bmp4* and *wnt5* and then assessed *slit* expression (S11 Fig). Inhibition of *bmp4* again resulted in reduced *slit* expression, particularly in the tail. Under these homeostatic RNAi conditions, inhibition of *wnt5* did not substantially alter *slit* expression but *slit*/*wnt5* reciprocal antagonism has been observed previously under regeneration conditions [28]. Double inhibition of both *bmp4* and *wnt5* resulted in reduced *slit* expression, similar to *bmp4* RNAi alone. Therefore, *wnt5* knockdown did not influence the reduced *slit* expression from *bmp4(RNAi)*, suggesting *bmp4* likely does not activate *slit* through repression of *wnt5*. One possible mechanism could be that graded *bmp4* activity activates midline *slit* expression at the highest point in the gradient at the dorsal midline and suppresses *wnt5* medially, while low levels of *bmp4* at the lateral edge enable expression of *wnt5*.

We last tested for possible ways that *slit* or *wnt5* could participate in control of AP axis information by controlling *wnt1*. However, under homeostatic RNAi conditions, *slit* or *wnt5* inhibition did not have a detectable effect on AP or ML expression of the *wnt1* posterior domain (S12 Fig). These data suggest *slit* and *wnt5* may act independently from the *bmp4*-dependent control mechanism regulating *wnt1’s* AP distribution under normal homeostatic conditions.

## Discussion

**Fig 6:**
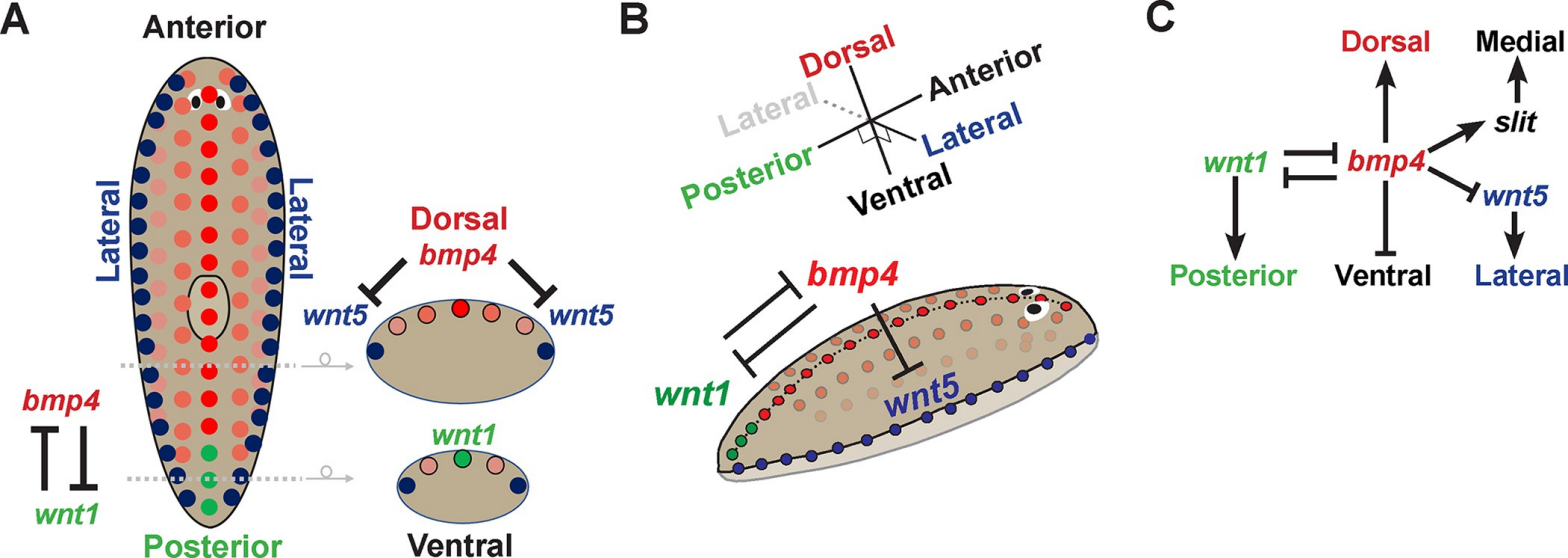
Model for homeostatic regulation of DV, AP, and ML axes by *bmp4*. **(A-B)** 2D and 3D models illustrating mutual antagonism between *bmp* and *wnt1* in the far posterior, *bmp4* inhibition of *wnt5* for control of lateral identity, and expression of key components across the AP and DV axes. Not shown, *bmp4* activates *slit* expression on the midline, *nog1;nog2* negatively regulate *bmp4* to promote *wnt1* posteriorly, and *bmp4* regulates dorsal-versus-ventral tissue identity.**(C)** Regulatory model highlighting key interactions connecting axis information in homeostatic animals. *bmp4* controls dorsal versus ventral identity, undergoes mutual inhibition with *wnt1* for control of posterior identity, and activates *slit* and represses *wnt5* in regulation mediolateral information.

Together, these experiments identify critical roles for *bmp4* in patterning multiple body axes in planarians. BMP regulation not only establishes DV polarity but additionally influences both posterior identity through regulation of *wnt1* and also ML polarity through the suppression of *wnt5* and activation of *slit*. These results contribute to prior work analyzing roles for *bmp4* and *slit/wnt5* on the regenerating anterior pole [37] to argue that major body patterning systems interact homeostatically in order to coordinate growth. We suggest that a regulatory logic in which the interaction of information across axes may be important for robustness of patterning across long timescales in adulthood (Fig 6).

The cross-regulation we observe between BMP, Wnt1 and Wnt5 signals in overall animal domains raises the question of how signal integration is achieved. Whether inputs from distinct axes can be resolved by individual or collections of the muscle cells involved in planarian positional information is an important and unresolved question for future analysis.

Our results also clarify the relationships among the BMP, Wnt5, and Slit signals that participate in ML patterning in planarians. DV polarity and dorsal *bmp4* expression were overall apparently normal in *wnt5(RNAi)* animals, although we found sparse *bmp4-*expressing cells to appear ventrally in such animals (S9 Fig) [28]. Although these RNAi conditions successfully knocked down *wnt5* and the inhibition caused ectopic lateral eye formation, it is possible that more complete inhibition of Wnt5 signaling could further impact the DV axis. However, with the present analysis, we find a much stronger effect of *bmp4* inhibition on *wnt5* expression than vice versa (Fig 5B). Furthermore, the double RNAi experiments testing *bmp4;wnt5(RNAi)* presented here suggest *wnt5* acts downstream of *bmp4* in eye placement as tested in these conditions (Fig 5D). These results argue strongly against a hypothetical model in which *wnt5* acts exclusively upstream of *bmp4*. Because of the above caveats, we cannot rule out the existence of *wnt5/bmp4* mutual antagonism, similar to that detected between *wnt1* and *bmp4*. However, our present results are more consistent with a mechanism for DV and ML formation in which *bmp4* has a primary role to delineate a *wnt5* expression domain which then exerts several effects, for example on ML eye placement, in a primarily downstream fashion. It has been previously demonstrated that BMP inhibits Wnt5 during ectoderm patterning in sea urchins [52], and BMP activity downregulates *wnt5a* to modulate convergence and extension in zebrafish development [53]. Therefore, roles of BMP upstream of Wnt5 factors may be conserved. *slit* also likely acts downstream of *bmp4*, because *bmp4* inhibition reduced *slit* expression. These results collectively argue that *bmp4* acts at a high point in a hierarchy for ML patterning.

Our results also identify mutual antagonism between posterior *wnt1* and dorsal *bmp4* expression domains in the posterior. *bmp4* also regulated the expression domain sizes of several posterior Wnt and Wnt-related factors. We found that in some cases, *bmp4* RNAi caused expression of posterior determinants to extend much further anterior than the tail (Figs 2 and 3). However, *bmp4(RNAi)* animals had normal tail size and pharynx placement (S6B Fig). On the other hand, our analysis of pharynx cell precursor markers *foxA* and *dd554* indicates *bmp4* indeed exerts effects on the distributions of AP-regionalized progenitors (S6A Fig). The relatively slow turnover of the pharynx may prevent identification of the consequences to organ position and/or maintenance in these experiments. In addition, *bmp4(RNAi)* animals ultimately underwent displacement of the pre-existing eyes, suggestive of complex shifts in AP identity (S6C Fig). However, the complex nature of these phenotypes suggests that a full understanding of how *bmp4* couples with Wnt signals to regulate AP tissue determination will likely require additional markers and assays of progenitor behavior and potentially ways to selectively impact *bmp4*’s AP regulatory functions.

Our results identifying roles of BMP on the AP axis are congruous with evidence of spatiotemporal coordination between the AP and DV axes in vertebrate development. In early mammalian development, a proximal BMP signal from trophectoderm activates Wnt3 asymmetrically in epiblast cells in order to establish the future AP axis [54]. In early *Xenopus* development, BMP4 is required for the expression of posterior Wnt8 [55]. The integration of BMP and Wnt signaling pathways occurs by Wnt8 preventing the conserved ability of GSK3 kinase to inhibit Smad1 activity through its phosphorylation and targeted proteasomal degradation, such that Wnt signals can positively enhance BMP signaling duration. This regulation contributes to a model in which AP positional information is specified by the duration of BMP signaling [56]. Zebrafish development also involves coordination of AP and DV axis formation, with maternal Wnt signaling generating the organizer to repress *bmp*, and zygotic Wnt transcriptionally promoting the maintenance of BMP expression [57]. Furthermore, BMP defines DV patterning in a temporal AP gradient from head to tail [58]. By contrast, planarian Wnt/BMP integration involves signaling antagonism and operates at a transcriptional level and so likely occurs through a distinct mechanism. Uses for Wnt and BMP signals to pattern perpendicular body axes predate the bilaterians, with Cnidarians using Wnt signaling along the oral-aboral primary body axis and BMP to control the perpendicular directive axis [3,12,59,60]. In *Nematostella*, BMP and canonical Wnt signaling mutually antagonize to pattern endomesoderm [61], reminiscent of our identification of mutual antagonism between planarian *wnt1* and *bmp4*. Our results suggest that interactions between BMP/Wnt signaling across axes may be a fundamental and ancient property enabling the integration of body axis information in three dimensions.

## Materials and methods

### Ethics statement

Procedures with *Schmidtea mediterranea* planarians (invertebrates) were conducted according to safety and ethics procedures in line with the Northwestern Office for Research Safety, with authorization from the Institutional Biosafety Committee (IBC), approved 12/7/21. As invertebrate animal subjects, planarians are not subject to IACUC or IRB review.

### Experimental model

Asexual *Schmidtea mediterranea* animals (CIW4 strain) were kept in 1x Montjuic salts (1.6 mmol/l NaCl, 1.0 mmol/l CaCl2, 1.0 mmol/l MgSO4, 0.1 mmol/l MgCl2, 0.1 mmol/l KCl and 1.2 mmol/l NaHCO3 prepared in Milli-Q water) at 18–20°C. Animals were fed pureed calf liver from aliquots stored at -80°C once a week and washed twice a week. Animals were starved at least 7 days before the start of experiments.

### Fluorescence in situ hybridization (FISH)

The FISH protocol used is based off previously published work [40]. Animals were treated with 7.5% NAC (w/v) in 1X PBS, fixed in 4% formaldehyde (w/v), and stored in methanol. They were rehydrated with methanol: 1x PBSTx, bleached in 6% hydrogen peroxide (v/v) in 1x PBS, permeabilized with proteinase K (20 mg/ml), then prehybridized at 56°C. Hybridization occurred with digoxigenin or fluorescein labeled riboprobes at a 1:1000 concentration (v/v), which were synthesized using T7 RNA binding sites for antisense transcription. Animals were washed in a SSC concentration series of 1:1 Pre-hybridization buffer:2xSSC, 2xSSC, and 0.2x SSC at 56°C. Anti-digoxigenin-POD (Sigma/Roche) or anti-fluorescein-POD antibodies (Sigma/Roche) were in a solution of 1x TNTx/ 10% (v/v) horse serum/ 10% (v/v) Western Blocking Reagent (Roche) at a concentration of 1:2000 (v/v). Tyramide in 1x TNTx was utilized to develop and amplify the antibody signal. For double FISH, the enzymatic activity of tyramide reactions was inhibited by sodium azide (100mM). Nuclei were stained using 1:1000 Hoescht (v/v, Invitrogen) in 1x TNTx.

### RNA interference (RNAi)

RNAi treatments were performed by feeding with dsRNA (16% v/v), red food dye (4% v/v), and pureed liver (80% v/v). Worms of each condition were cultured in separate petri dishes in 1x Montjuic salts. dsRNA was synthesized as previously described [29]. For controls, the dsRNA synthesized was either *Photinus pyralis luciferase (Ppluc)* or *C*. *elegans unc-22*, as these genes are not present in the *Schmidtea mediterranea* genome. Animals were fed 1 ul RNAi food mix per worm every 2–3 days for the indicated length of the experiment. For double RNAi, control dsRNA was mixed in with the single experimental dsRNA to ensure the same amount of overall dsRNA in feedings between double and single experimental conditions. This resulted in a 1:1 dsRNA mix of *Ppluc*:*Ppluc* for control RNAi, *Ppluc*:*bmp4* for *bmp4* RNAi, *Ppluc*:*wnt5* for *wnt5* RNAi, and *bmp4*:*wnt5* as the double RNAi knockdown (Figs 5D, S10 and S11). For RNAi treatment without injury, animals were fixed 5 days after the last feeding. For a regeneration time course following injury, animals were cut 2 days after the last feeding and fixed at the indicated time.

### Image acquisition

Live animals were imaged with a Leica M210F dissecting microscope with a Leica DFC295 camera (Fig 5D). Stained animals were imaged with a Leica Stellaris confocal microscope using a 10x or 20x objective and Leica LAS-X software (Figs 1–5, S3, S4, S6–S9, S11 and S12). FISH images are maximum projections from a z-stack and representative of each condition. Unless otherwise specified, z-stacks were chosen to segment to approximately half way through the dorso-ventral axis from animals viewed either dorsally or ventrally as indicated. Adjustments to brightness and contrast were made using Adobe Photoshop or ImageJ.

### Irradiation

Animals were irradiated using a Radsource RS-2000 X-ray to deliver 1350 rads to worms in petri dishes of 1x Montjuic salts (S4 Fig). Following irradiation, the first RNAi feeding was administered.

### qRT-PCR expression analysis

RNA was purified from whole animals in trizol using a Turrex tissue homogenizer. Four biological replicates were generated for each RNAi condition. cDNA was synthesized using the Multiscribe reverse transcriptase kit (Applied Biosystems) following DNase treatment (Turbo DNA-free, Ambion). Control samples were generated by exclusion of reverse transcription reagents. cDNA was treated with 40% RNAse H (v/v, New England Biosciences) then diluted 1:5. The QuantStudio3 QPCR system and SYBR green (Roche KAPA biosystems) were used for analysis (S10 Fig). Detection of *bmp4* and *wnt5* mRNA was normalized to reference gene *ubiquilin* and relative expression values calculated using the delta-delta Ct method. Detection of mRNA abundance was validated by comparison between Ct values in treatments that either included or excluded the reverse transcription enzyme, and mRNA signal was evaluated for detection at least 8-fold above background as averaged across all samples. Outliers were flagged for removal by Grubb’s outlier test with alpha < 0.05 with respect to all Cts tested for each probe set. Sequences of the qRT-PCR primers used are included in S1 Table.

### Cloning

Primers for dsRNA, riboprobes, and qRT-PCR are listed in S1 Table. Riboprobes for *foxD*, *dd554*, and *porcupine* were described previously [48,62,63]. Briefly, RNA was isolated from adult animals and fragments undergoing regeneration in a mixed-stage timeseries using Trizol and a Turrex tissue homogenizer, followed by ethanol precipitation and DNAse treatment (DNA-free, Ambion). Reverse transcription was performed using Superscript III and oligo-dT priming. Primers were designed using Primer3 (https://primer3.ut.ee/) and planarian sequences analyzed from Planmine (https://planmine.mpinat.mpg.de/planmine/begin.do) [64]. Planarian cDNAs were cloned by PCR using the primers indicated in S1 Table and either cloned into pGem-T-easy or directly used for PCR-mediated addition of T7 sequences to 3’ ends for riboprobe synthesis or to 5’ and 3’ ends for dsRNA synthesis.

### Quantification and statistical analysis

Body length and PCG measurements were taken using Leica LAS X or FIJI on z-stack maximum projections of FISH images. For body length, animals were measured from their most anterior to most posterior tips. For PCG length, animals were measured from the tip of the anterior (Figs 3B, S5 and S6C), tip of the posterior (Figs 2, 3A, 3C, S2A, S2C–S2E, S3 and S12), or lateral edge (Figs 5B, 5C, S2B and S8A) to end of the fluorescence stain. For measuring head, pharynx, and tail lengths, animals were measured from anterior tip to the start of the *dd554* stain, the length of the *dd554* stain, the end of the *dd554* stain to the posterior tip, respectively (S6B Fig). Cell counting was manually performed using maximum projections in LAS X (Figs 1, S2, S5 and S8A). Line-scan intensity measurements of AP distributions of *bmp4* and *wnt1* were performed in FIJI along the midline using line thickness of 30 pixels for measurements, which corresponded to ~1/10 of the width of the animal measured at the pharynx region (Figs 4 and S1A). Measurements of *wnt1*, *bmp4*, and *dd23400* ML expression were taken using line thickness of 120 pixels, which corresponded to ~1/10 the length of the animal (S1B Fig). Cartoons indicate AP position of measured regions. Measurements were normalized by length along each region, and by maximum intensity across the scan, then summed normalized intensities were computed across equal-sized bins and plotted in R Studio (S1 Fig) or Microsoft Excel (Fig 4). Box plots were generated in BoxPlotR. Statistical analysis was conducted using Microsoft Excel or R. A student’s t-test was used for comparison between the means of two populations (Excel). One-way ANOVA on ranks (Kruskal-Wallis test) and Dunnett’s test were used for comparison between the means of multiple populations (R Studio). Data used to generate figures is included in S1 Data.

## Supporting information

S1 Fig Expression intensity of *bmp4* and *wnt1* across the ML and AP axes. Analysis of normalized FISH line-scan intensity from dorsal-view images represented in Fig 1A measuring **(A)** mediolateral expression of *bmp4* and *dd23400* from an anterior tail domain, **(B)** mediolateral expression of *dd23400* and *wnt1* measured from a posterior tail domain, and **(C)** anteroposterior expression of *wnt1* and *bmp4* measured within the tail. 3 biological replicate images were processed in ImageJ to measure line-scan intensity with a width of 120 (A-B) or 30 pixels (C), encompassing approximately 1/10^th^ the size of the orthogonal axis. Cartoons indicate the regions and approximate relative scale of measurements taken for each analysis (R1, R2, R3). Data were position-normalized and intensity-normalized, then summed within 15 equal-sized bins dividing each region. Averages and standard deviations across biological replicates for each bin are presented in each plot (A-C). Statistical tests were performed to assess overall expression trends using t-tests to compare sample intensities across the bins indicated by the dotted lines. t-test for ML-*bmp4* distributions compares data from bins 1–3 versus bins 7–9 (N = 9 measurements for each region), t-tests for ML plots of *dd23400* and *wnt1* compares data from bins 4–6 versus bins 7–9 (N = 9 measurements for each region), and t-tests for AP *bmp4* and *wnt1* compares data from bins 1–4 versus bins 5–8 (N = 12 measurements for each region). **(D-F)** Plots of individual biological replicates after the normalization procedure described above. The key trends observed in this analysis are that *bmp4* expression is present in a graded fashion on the mediolateral axis dorsally, *dd23400* is expressed sharply at the midline, *wnt1* is expressed at the midline in the posterior, and *bmp4* expression along the posterior midline reduces in the far posterior at approximately the same location as *wnt1* is expressed.
(PDF)

S2 Fig Quantification of BMP signaling on *wnt1*+ cells. **(A-E**) Quantification of absolute and relative *wnt1+* cell numbers after inhibition of indicated genes related to Fig 2. Graphs showing absolute numbers of *wnt1+* cells and numbers of *wnt1+* cells normalized to body length for **(A)** 14 days of control or *bmp4* RNAi, **(C)** 18 days of control or *nog1;nog2* RNAi, **(D)** 14 days of control, *smad1*, or *smad4* RNAi, or **(E)** 18 days of control, *tbx2/3*, or *nlg8* RNAi. **(B)** Graph illustrating the maximum width between *wnt1+* cells relative to animal body width following 14 days of control or *bmp4* RNAi. **(A-E)** N ≥ 4 animals. Plots shows median values (middle bars) and first-to-third interquartile ranges (boxes); whiskers indicate 1.5× the interquartile ranges and dots are data points from individual animals. *p<0.05, **p<0.01, ****p<0.0001, n.s. indicates p>0.05 by 2-tailed t-test (A-C) or by one-way ANOVA on ranks (D-E).
(PDF)

S3 Fig Reduction of *wnt1* expression domain through AP regenerative rescaling is not influenced by *bmp4* inhibition. **(A)** FISH to detect *wnt1* expression in regenerating tail fragments at 0, 18, and 96 hours after amputations conducted after 14 days of either control or *bmp4* RNAi. Arrows indicate anterior-most *wnt1+* cell detected along the dorsal midline for each timepoint and condition. Top panels show control animals undergoing early expansion of the dorsal midline *wnt1* domain by 18 hours of regeneration followed by rescaling to reduce the domain to the tip of the animal by 96 hours. Bottom panel shows *wnt1* expression dynamics in *bmp4* RNAi, in which midline *wnt1* expression was anterior expanded at the time of injury (0 hours), remained expanded at 18 hours of regeneration, and then restricted posteriorly by 96 hours, similar to control RNAi conditions. Therefore, BMP pathway modulation is unlikely to be responsible for the normal restriction of *wnt1* by 96 hours in regenerating tail fragments. Scale bars represent 150 μm. **(B)** Graph showing the quantification of the length of the *wnt1* domain relative to length of tail fragment. ****p<0.0001 by 2-tailed t-test and n.s. indicates p>0.05; N ≥ 3 animals. Box plots shows median values (middle bars) and first to third interquartile ranges (boxes); whiskers indicate 1.5× the interquartile ranges and dots are data points from individual animals.
(PDF)

S4 Fig New cell turnover is required for *wnt1* domain expansion following *bmp4* RNAi. FISH detecting *wnt1* in control and *bmp4(RNAi)* animals given 5 dsRNA dosings over 12 days following either no irradiation or given a sublethal dose of 1350 rads of X-ray irradiation. Unirradiated *bmp4(*RNAi) animals underwent expansion of *wnt1* expression compared to unirradiated control RNAi conditions. By contrast, *wnt1* expression was not present in irradiated control or *bmp4(RNAi)* animals. N ≥ 5. Scale bars represent 300 μm.
(PDF)

S5 Fig Inhibition of *bmp4* does not alter number of anterior pole *notum+* cells. Plots of absolute and size-normalized numbers of anterior pole *notum+* cells following homeostatic inhibition of *bmp4* RNAi versus control RNAi animals, related to Fig 3B. Cells were manually scored from dorsal-view images obtained at 20x. Knockdown of *bmp4* did not significantly change the absolute or bodysize-relative number of *notum+* cells. n.s. indicates p>0.05 by 2-tailed t-test. N ≥ 6 animals. Plots shows median values (middle bars) and first-to-third interquartile ranges (boxes); whiskers indicate 1.5× the interquartile ranges and dots are data points from individual animals.
(PDF)

S6 Fig Effects of *bmp4* inhibition on markers of differentiated tissues and progenitors. **(A)** Following 28 days RNAi of control or *bmp4* RNAi, animals were stained for gut marker *porcupine* and pharynx-associated markers *SMU15007112*, *dd554*, *foxA*, and *laminin*. Inhibition of *bmp4* did not affect *porcupine* or *SMU15007112* expression. *bmp4* RNAi caused a subtle change to *dd554* expression which kept overall pharynx staining intact but eliminated expression of streams of *dd554+* cells located outside of the pharynx (zoom-ins, 6/7 animals had no *dd554* expressing cells adjacent to the pharynx, compared with 7/7 control animals displaying this expression). However, inhibition of *bmp4* consistently resulted in ectopic non-pharynx expression of *foxA* and ectopic anterior expression of *laminin*. 5/8 *bmp4(RNAi)* animals had relatively less reduction to *laminin* pharynx staining and less ectopic anterior *laminin* and 3/8 animals had coincident loss of *laminin* expression and stronger gain of ectopic anterior expression. N ≥ 7. Scale bars represent 300 μm. **(B)** Measurements of head, pharynx, and tail regions determined by *dd554* expression relative to body region for control and *bmp4* RNAi. *bmp4* inhibition did not significantly alter AP pharynx scale or position or size of tail and anterior (head) domain as measured with respect to pharynx position. **(C)** Left: Hoechst-stained animals following 28 days of control or *bmp4* RNAi to detect original and ectopic eyes. Right: Quantification of the length from the anterior tip of the eyes (as measured for each pair of eyes along the midline) and normalized to total body size. Top panels show zoom-in of anterior animal regions for control and *bmp4(RNAi)* animals of eyes. Images are max-projections with z-planes chosen to show only either the original or ectopic *bmp4(RNAi)* animal eyes as indicated. Compared to the position of control eyes, original eyes in *bmp4(RNAi)* animals were displaced anteriorly toward the tip of the head while ectopic eyes are positioned at the same relative location as normal eyes in control animals. Scale bars represent 300 um. **(B-C)** ****p<0.0001, n.s. indicates p>0.05 by 2-tailed t-test (B) or one way ANOVA test on ranks (C). N = 7 animals. Plots shows median values (middle bars) and first-to-third interquartile ranges (boxes); whiskers indicate 1.5× the interquartile ranges and dots are data points from individual animals.
(PDF)

S7 Fig Additional data testing roles of *wnt1*, *nog1+nog2*, and *notum* on *bmp* expression in the tail. **(A)** FISH staining for *bmp4* expression in whole animals after 14 days of control or *wnt1* RNAi related to Fig 4. Inhibition of *wnt1* resulted in elevated expression of *bmp4* on the posterior midline of the animal (arrows) and did not change the lack of ventral *bmp4* expression (right). Scale bars are 150 μm. N = 21 animals. **(B)** Double FISH of control or *wnt1* RNAi for *bmp4* and anterior marker *foxD*. N = 8. Inhibition of *wnt1* caused elevated expression of *bmp4* in the posterior but did not alter the lack of posterior *foxD*, suggesting *wnt1*’s role on *bmp4* expression is not likely due to control of head-versus-tail identity determination. **(C)** Animals stained for *bmp4* following control or *notum* RNAi homeostatically for 14 days. Inhibition of *notum* did not alter *bmp4* expression on either the dorsal or ventral side of the animals. Scale bars are 300 μm. N ≥ 6. **(D)** Dorsal posterior view of *bmp4* FISH conducted on control or *nog1;nog2(RNAi)* animals inhibited homeostatically for 18 days. *nog1;nog2* inhibition qualitatively appeared to cause an increase in overall *bmp4* expression levels. N = 8. Scale bars are 150 μm.
(PDF)

S8 Fig *bmp4* promotes midline identity and suppresses lateral identity. **(A-C)** FISH for *dd23400*, *LaminB*, and *wnt5* following 28 days of control or *bmp4* RNAi. Scale bars represent 300 μm. **(A)** Inhibition of *bmp4* reduces *dd23400* expression (arrows), particularly in the posterior. Bottom Left: Quantification of number of *dd23400+* cells normalized to animal body length. Bottom Right: Quantification of maximum width between *dd23400+* cells relative to animal body width. *p<0.05, **p<0.01 by unpaired 2-tailed t-test; N ≥ 6 animals. Box plots show median values (middle bars) and first to third interquartile ranges (boxes); whiskers indicate 1.5× the interquartile ranges and dots are data points from individual animals. **(B)** *bmp4* RNAi causes ectopic medial expression of lateral marker *laminB* expression on the posterior midline (arrows). **(C)** Knockdown of *bmp4* appears to elevate *wnt5* expression less dramatically on the ventral side versus dorsal side. Right panels show enlargements of boxed regions.
(PDF)

S9 Fig *wnt5* inhibition does not detectably alter *bmp4*’s dorsal expression and causes weak *bmp4* expression ventrally. FISH staining for *bmp4* after 21 days of control or *wnt5* RNAi under homeostatic conditions. Inhibition of *wnt5* does not cause detectable increases or decreases in *bmp4* expression or distribution on the dorsal side of animals. However, some ectopic ventral expression appears following *wnt5* RNAi. Scale bars are 150 μm. N = 5 animals.
(PDF)

S10 Fig Verification of *bmp4;wnt5* knockdown. Left: qPCR to detect expression of *bmp4* transcript (normalized to *ubiquilin* control) to detect knockdown of *bmp4* in *bmp4* RNAi and *bmp4;wnt5* RNAi. Animals were treated with dsRNA for 20 days (9 dsRNA feedings), followed by isolation of RNA, followed by RT-qPCR. *bmp4* expression was knocked down after delivery of either *bmp4* dsRNA or the combination of *bmp4* and *wnt5* dsRNA. Right: qPCR to detect expression of *wnt5* after RNAi of *wnt5* individually or in combination with *bmp4* under the same conditions. Both single and double RNAi conditions caused significant *wnt5* knockdown. *p<0.05, **p<0.01 by one-tailed t-test to determine if mRNA reduced after RNAi. Plots show median values (middle bars) and first-to-third interquartile ranges (boxes); whiskers indicate 1.5× the interquartile ranges and dots are data points from individual animals.
(PDF)

S11 Fig *wnt5* co-inhibition does not rescue the *bmp4(RNAi)* phenotype of reduced *slit* expression. FISH staining for *slit* following 20 days of control, *bmp4*, *wnt5*, and *bmp4;wnt5* RNAi. Loss of *bmp4* reduces *slit* expression (arrows). Loss of *wnt5* did not appear to alter *slit* expression. Knockdown of both *bmp4* and *wnt5* resulted in the reduced *slit* expression, similar to *bmp4* RNAi (arrows). N ≥ 6 animals. Scale bars represent 300 μm.
(PDF)

S12 Fig Homeostatic inhibition of *slit* or *wnt5* does not alter *wnt1* expression. Left: Following 18 days of control, *bmp4*, *slit*, or *wnt5* RNAi, animals were stained for *wnt1* expression. Inhibition of *bmp4* expanded *wnt1* anteriorly and reduced posterior expression. Inhibition of *slit* or *wnt5* did not significantly affect *wnt1* expression. N ≥ 6 animals. Scale bars represent 300 μm. Right: Plot showing length of *wnt1* domain from the tip of the tail relative to animal body length. n.s. indicates p>0.05, ****p<0.0001 by one-way ANOVA on ranks. N ≥ 8 animals. Plots show median values (middle bars) and first-to-third interquartile ranges (boxes); whiskers indicate 1.5× the interquartile ranges and dots are data points from individual animals.
(PDF)

S1 Table Primer Sequences for dsRNA, riboprobes, and qRT-PCR. (PDF)

S1 Data Data used for plotting figures. (XLSX)

## References

[1] Petersen CP, Reddien PW. Wnt signaling and the polarity of the primary body axis. Cell. 2009;139(6):1056–68. doi: 10.1016/j.cell.2009.11.035 20005801

[2] De Robertis EM, Sasai Y. A common plan for dorsoventral patterning in Bilateria. Nature. 1996;380(6569):37–40. doi: 10.1038/380037a0 8598900

[3] Niehrs C. On growth and form: a Cartesian coordinate system of Wnt and BMP signaling specifies bilaterian body axes. Development. 2010;137(6):845–57. doi: 10.1242/dev.039651 20179091

[4] Roth S, Neuman-Silberberg FS, Barcelo G, Schupbach T. cornichon and the EGF receptor signaling process are necessary for both anterior-posterior and dorsal-ventral pattern formation in Drosophila. Cell. 1995;81(6):967–78. doi: 10.1016/0092-8674(95)90016-0 7540118

[5] Rossant J, Tam PP. Blastocyst lineage formation, early embryonic asymmetries and axis patterning in the mouse. Development. 2009;136(5):701–13. doi: 10.1242/dev.017178 19201946

[6] Molina MD, Salo E, Cebria F. The BMP pathway is essential for re-specification and maintenance of the dorsoventral axis in regenerating and intact planarians. Developmental biology. 2007;311(1):79–94. doi: 10.1016/j.ydbio.2007.08.019 17905225

[7] Gurley KA, Rink JC, Sanchez Alvarado A. Beta-catenin defines head versus tail identity during planarian regeneration and homeostasis. Science. 2008;319(5861):323–7. doi: 10.1126/science.1150029 18063757 PMC2755502

[8] Iglesias M, Gomez-Skarmeta JL, Saló E, Adell T. Silencing of Smed-betacatenin1 generates radial-like hypercephalized planarians. Development. 2008;135(7):1215–21. doi: 10.1242/dev.020289 18287199

[9] Petersen CP, Reddien PW. Smed-betacatenin-1 is required for anteroposterior blastema polarity in planarian regeneration. Science. 2008;319(5861):327–30. doi: 10.1126/science.1149943 18063755

[10] Reddien PW, Bermange AL, Kicza AM, Sanchez Alvarado A. BMP signaling regulates the dorsal planarian midline and is needed for asymmetric regeneration. Development. 2007;134(22):4043–51. doi: 10.1242/dev.007138 17942485

[11] Srivastava M, Mazza-Curll KL, van Wolfswinkel JC, Reddien PW. Whole-body acoel regeneration is controlled by Wnt and Bmp-Admp signaling. Curr Biol. 2014;24(10):1107–13. doi: 10.1016/j.cub.2014.03.042 24768051

[12] Holstein TW. The role of cnidarian developmental biology in unraveling axis formation and Wnt signaling. Developmental biology. 2022;487:74–98. doi: 10.1016/j.ydbio.2022.04.005 35461834

[13] Reddy PC, Gungi A, Ubhe S, Pradhan SJ, Kolte A, Galande S. Molecular signature of an ancient organizer regulated by Wnt/β-catenin signalling during primary body axis patterning in Hydra. Communications Biology. 2019;2(1):434. 31799436 10.1038/s42003-019-0680-3 PMC6879750

[14] Fincher CT, Wurtzel O, de Hoog T, Kravarik KM, Reddien PW. Cell type transcriptome atlas for the planarian Schmidtea mediterranea. Science. 2018;360(6391):eaaq1736. doi: 10.1126/science.aaq1736 29674431 PMC6563842

[15] Plass M, Solana J, Wolf FA, Ayoub S, Misios A, Glazar P, et al. Cell type atlas and lineage tree of a whole complex animal by single-cell transcriptomics. Science. 2018;360(6391). doi: 10.1126/science.aaq1723 29674432

[16] Wurtzel O, Oderberg IM, Reddien PW. Planarian Epidermal Stem Cells Respond to Positional Cues to Promote Cell-Type Diversity. Dev Cell. 2017;40(5):491–504.e5. doi: 10.1016/j.devcel.2017.02.008 28292427 PMC5679284

[17] Lapan SW, Reddien PW. Transcriptome analysis of the planarian eye identifies ovo as a specific regulator of eye regeneration. Cell Rep. 2012;2(2):294–307. doi: 10.1016/j.celrep.2012.06.018 22884275 PMC3785364

[18] Adler CE, Seidel CW, McKinney SA, Sánchez Alvarado A. Selective amputation of the pharynx identifies a FoxA-dependent regeneration program in planaria. eLife. 2014;3:e02238. doi: 10.7554/eLife.02238 24737865 PMC3985184

[19] Bonar NA, Petersen CP. Integrin suppresses neurogenesis and regulates brain tissue assembly in planarian regeneration. Development. 2017;144(5):784–94. doi: 10.1242/dev.139964 28126842 PMC5374345

[20] Seebeck F, März M, Meyer A-W, Reuter H, Vogg MC, Stehling M, et al. Integrins are required for tissue organization and restriction of neurogenesis in regenerating planarians. Development. 2017;144(5):795–807. doi: 10.1242/dev.139774 28137894 PMC5374344

[21] Abnave P, Aboukhatwa E, Kosaka N, Thompson J, Hill MA, Aboobaker AA. Epithelial-mesenchymal transition transcription factors control pluripotent adult stem cell migration in vivo in planarians. Development. 2017;144(19):3440–53. doi: 10.1242/dev.154971 28893948 PMC5665486

[22] Guedelhoefer OCt, Sánchez Alvarado A. Amputation induces stem cell mobilization to sites of injury during planarian regeneration. Development. 2012;139(19):3510–20. doi: 10.1242/dev.082099 22899852 PMC3436109

[23] Atabay KD, LoCascio SA, de Hoog T, Reddien PW. Self-organization and progenitor targeting generate stable patterns in planarian regeneration. Science. 2018;360(6387):404–9. doi: 10.1126/science.aap8179 29545509 PMC6135251

[24] Lander R, Petersen CP. Wnt, Ptk7, and FGFRL expression gradients control trunk positional identity in planarian regeneration. eLife. 2016;5:e12850. doi: 10.7554/eLife.12850 27074666 PMC4865369

[25] Hill EM, Petersen CP. Positional information specifies the site of organ regeneration and not tissue maintenance in planarians. eLife. 2018;7:e33680. doi: 10.7554/eLife.33680 29547123 PMC5866098

[26] Witchley JN, Mayer M, Wagner DE, Owen JH, Reddien PW. Muscle cells provide instructions for planarian regeneration. Cell Rep. 2013;4(4):633–41. doi: 10.1016/j.celrep.2013.07.022 23954785 PMC4101538

[27] Petersen CP, Reddien PW. A wound-induced Wnt expression program controls planarian regeneration polarity. Proceedings of the National Academy of Sciences of the United States of America. 2009;106(40):17061–6. doi: 10.1073/pnas.0906823106 19805089 PMC2743725

[28] Gurley KA, Elliott SA, Simakov O, Schmidt HA, Holstein TW, Sanchez Alvarado A. Expression of secreted Wnt pathway components reveals unexpected complexity of the planarian amputation response. Developmental biology. 2010;347(1):24–39. doi: 10.1016/j.ydbio.2010.08.007 20707997 PMC2966944

[29] Petersen CP, Reddien PW. Polarized notum activation at wounds inhibits Wnt function to promote planarian head regeneration. Science. 2011;332(6031):852–5. doi: 10.1126/science.1202143 21566195 PMC3320723

[30] Sureda-Gómez M, Pascual-Carreras E, Adell T. Posterior Wnts Have Distinct Roles in Specification and Patterning of the Planarian Posterior Region. Int J Mol Sci. 2015;16(11):26543–54. doi: 10.3390/ijms161125970 26556349 PMC4661829

[31] Adell T, Salò E, Boutros M, Bartscherer K. Smed-Evi/Wntless is required for beta-catenin-dependent and -independent processes during planarian regeneration. Development. 2009;136(6):905–10. doi: 10.1242/dev.033761 19211673

[32] Gavino MA, Reddien PW. A Bmp/Admp regulatory circuit controls maintenance and regeneration of dorsal-ventral polarity in planarians. Current biology: CB. 2011;21(4):294–9. 21295483 10.1016/j.cub.2011.01.017 PMC3079492

[33] Molina MD, Neto A, Maeso I, Gomez-Skarmeta JL, Salo E, Cebria F. Noggin and noggin-like genes control dorsoventral axis regeneration in planarians. Current biology: CB. 2011;21(4):300–5. 21295481 10.1016/j.cub.2011.01.016

[34] Orii H, Watanabe K. Bone morphogenetic protein is required for dorso-ventral patterning in the planarian Dugesia japonica. Development, growth & differentiation. 2007;49(4):345–9. doi: 10.1111/j.1440-169X.2007.00931.x 17501910

[35] Gonzalez-Sastre A, Molina MD, Salo E. Inhibitory Smads and bone morphogenetic protein (BMP) modulate anterior photoreceptor cell number during planarian eye regeneration. The International journal of developmental biology. 2012;56(1–3):155–63. doi: 10.1387/ijdb.123494ag 22451003

[36] Cebrià F, Guo T, Jopek J, Newmark PA. Regeneration and maintenance of the planarian midline is regulated by a slit orthologue. Dev Biol. 2007;307(2):394–406. doi: 10.1016/j.ydbio.2007.05.006 17553481 PMC2148499

[37] Oderberg IM, Li DJ, Scimone ML, Gavino MA, Reddien PW. Landmarks in Existing Tissue at Wounds Are Utilized to Generate Pattern in Regenerating Tissue. Current biology: CB. 2017;27(5):733–42. 28216315 10.1016/j.cub.2017.01.024 PMC5801735

[38] Scimone ML, Cloutier JK, Maybrun CL, Reddien PW. The planarian wound epidermis gene equinox is required for blastema formation in regeneration. Nature communications. 2022;13(1):2726. doi: 10.1038/s41467-022-30412-6 35585061 PMC9117669

[39] Orii HK K; Agata K; Watanabe K. Molecular Cloning of the Bone Morphogenetic Protein (BMP) Gene from the Planarian Dugesia japonica. Zoological science. 1998;15(6):6.

[40] Schad EG, Petersen CP. STRIPAK Limits Stem Cell Differentiation of a WNT Signaling Center to Control Planarian Axis Scaling. Curr Biol. 2020;30(2):254–63.e2. doi: 10.1016/j.cub.2019.11.068 31928872 PMC7153782

[41] Tian Q, Sun Y, Gao T, Li J, Hao Z, Fang H, et al. TBX2/3 is required for regeneration of dorsal-ventral and medial-lateral polarity in planarians. J Cell Biochem. 2021;122(7):731–8. doi: 10.1002/jcb.29905 33586232

[42] Scimone ML, Cote LE, Rogers T, Reddien PW. Two FGFRL-Wnt circuits organize the planarian anteroposterior axis. eLife. 2016;5. doi: 10.7554/eLife.12845 27063937 PMC4865367

[43] Stückemann T, Cleland JP, Werner S, Thi-Kim Vu H, Bayersdorf R, Liu SY, et al. Antagonistic Self-Organizing Patterning Systems Control Maintenance and Regeneration of the Anteroposterior Axis in Planarians. Dev Cell. 2017;40(3):248–63.e4. doi: 10.1016/j.devcel.2016.12.024 28171748

[44] Tewari AG, Owen JH, Petersen CP, Wagner DE, Reddien PW. A small set of conserved genes, including sp5 and Hox, are activated by Wnt signaling in the posterior of planarians and acoels. PLoS Genet. 2019;15(10):e1008401. doi: 10.1371/journal.pgen.1008401 31626630 PMC6821139

[45] Reuter H, Marz M, Vogg MC, Eccles D, Grifol-Boldu L, Wehner D, et al. Beta-catenin-dependent control of positional information along the AP body axis in planarians involves a teashirt family member. Cell Rep. 2015;10(2):253–65. 25558068 10.1016/j.celrep.2014.12.018

[46] Oderberg IM, Li DJ, Scimone ML, Gaviño MA, Reddien PW. Landmarks in Existing Tissue at Wounds Are Utilized to Generate Pattern in Regenerating Tissue. Curr Biol. 2017;27(5):733–42. doi: 10.1016/j.cub.2017.01.024 28216315 PMC5801735

[47] Cebria F, Newmark PA. Planarian homologs of netrin and netrin receptor are required for proper regeneration of the central nervous system and the maintenance of nervous system architecture. Development. 2005;132(16):3691–703. doi: 10.1242/dev.01941 16033796

[48] Zhu SJ, Hallows SE, Currie KW, Xu C, Pearson BJ. A mex3 homolog is required for differentiation during planarian stem cell lineage development. eLife. 2015;4. doi: 10.7554/eLife.07025 26114597 PMC4507787

[49] Fincher CT, Wurtzel O, de Hoog T, Kravarik KM, Reddien PW. Cell type transcriptome atlas for the planarian Schmidtea mediterranea. Science. 2018;360(6391). doi: 10.1126/science.aaq1736 29674431 PMC6563842

[50] Scimone ML, Kravarik KM, Lapan SW, Reddien PW. Neoblast specialization in regeneration of the planarian Schmidtea mediterranea. Stem cell reports. 2014;3(2):339–52. doi: 10.1016/j.stemcr.2014.06.001 25254346 PMC4176530

[51] Sureda-Gomez M, Pascual-Carreras E, Adell T. Posterior Wnts Have Distinct Roles in Specification and Patterning of the Planarian Posterior Region. International journal of molecular sciences. 2015;16(11):26543–54. doi: 10.3390/ijms161125970 26556349 PMC4661829

[52] McIntyre DC, Seay NW, Croce JC, McClay DR. Short-range Wnt5 signaling initiates specification of sea urchin posterior ectoderm. Development. 2013;140(24):4881–9. doi: 10.1242/dev.095844 24227654 PMC3848187

[53] Myers DC, Sepich DS, Solnica-Krezel L. Bmp activity gradient regulates convergent extension during zebrafish gastrulation. Dev Biol. 2002;243(1):81–98. doi: 10.1006/dbio.2001.0523 11846479

[54] Kurek D, Neagu A, Tastemel M, Tüysüz N, Lehmann J, van de Werken HJG, et al. Endogenous WNT signals mediate BMP-induced and spontaneous differentiation of epiblast stem cells and human embryonic stem cells. Stem Cell Reports. 2015;4(1):114–28. doi: 10.1016/j.stemcr.2014.11.007 25544567 PMC4297870

[55] Hoppler S, Moon RT. BMP-2/-4 and Wnt-8 cooperatively pattern the Xenopus mesoderm. Mech Dev. 1998;71(1–2):119–29. doi: 10.1016/s0925-4773(98)00004-5 9507084

[56] Fuentealba LC, Eivers E, Ikeda A, Hurtado C, Kuroda H, Pera EM, et al. Integrating patterning signals: Wnt/GSK3 regulates the duration of the BMP/Smad1 signal. Cell. 2007;131(5):980–93. doi: 10.1016/j.cell.2007.09.027 18045539 PMC2200633

[57] Tuazon FB, Mullins MC. Temporally coordinated signals progressively pattern the anteroposterior and dorsoventral body axes. Semin Cell Dev Biol. 2015;42:118–33. doi: 10.1016/j.semcdb.2015.06.003 26123688 PMC4562868

[58] Tucker JA, Mintzer KA, Mullins MC. The BMP signaling gradient patterns dorsoventral tissues in a temporally progressive manner along the anteroposterior axis. Dev Cell. 2008;14(1):108–19. doi: 10.1016/j.devcel.2007.11.004 18194657 PMC2266782

[59] Rentzsch F, Anton R, Saina M, Hammerschmidt M, Holstein TW, Technau U. Asymmetric expression of the BMP antagonists chordin and gremlin in the sea anemone Nematostella vectensis: implications for the evolution of axial patterning. Developmental biology. 2006;296(2):375–87. doi: 10.1016/j.ydbio.2006.06.003 16828077

[60] Saina M, Genikhovich G, Renfer E, Technau U. BMPs and Chordin regulate patterning of the directive axis in a sea anemone. Proceedings of the National Academy of Sciences. 2009;106(44):18592–7. 10.1073/pnas.0900151106 PMC2773963 19833871

[61] Wijesena N, Simmons DK, Martindale MQ. Antagonistic BMP-cWNT signaling in the cnidarian Nematostella vectensis reveals insight into the evolution of mesoderm. Proc Natl Acad Sci U S A. 2017;114(28):E5608–e15. doi: 10.1073/pnas.1701607114 28652368 PMC5514723

[62] Scimone ML, Lapan SW, Reddien PW. A forkhead transcription factor is wound-induced at the planarian midline and required for anterior pole regeneration. PLoS genetics. 2014;10(1):e1003999. doi: 10.1371/journal.pgen.1003999 24415944 PMC3886891

[63] Rink JC, Gurley KA, Elliott SA, Sanchez Alvarado A. Planarian Hh signaling regulates regeneration polarity and links Hh pathway evolution to cilia. Science. 2009;326(5958):1406–10. doi: 10.1126/science.1178712 19933103 PMC2861735

[64] Rozanski A, Moon H, Brandl H, Martin-Duran JM, Grohme MA, Huttner K, et al. PlanMine 3.0-improvements to a mineable resource of flatworm biology and biodiversity. Nucleic acids research. 2019;47(D1):D812–D20. doi: 10.1093/nar/gky1070 30496475 PMC6324014

