# Supplementary figures and images for: BMP suppresses WNT to integrate patterning of orthogonal body axes in adult planarians
Source: PLoS Genet. 2023 Sep 20;19(9):e1010608. doi: 10.1371/journal.pgen.1010608 (PMC10545109; doi:10.1371/journal.pgen.1010608)

**A**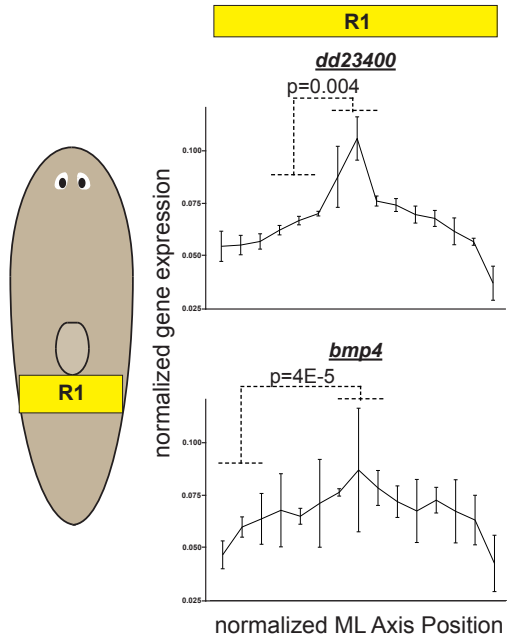**B**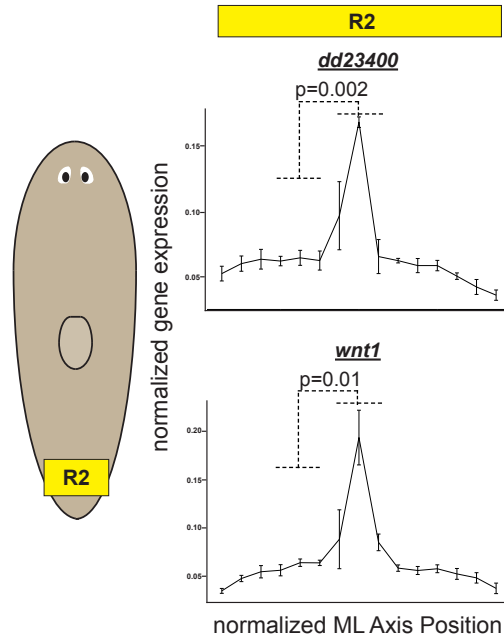**C**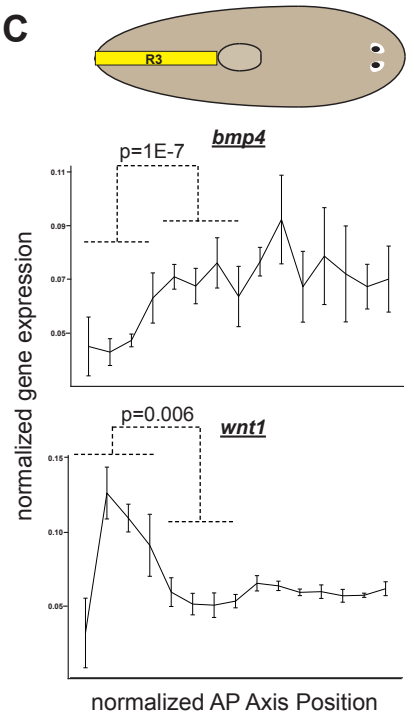**D**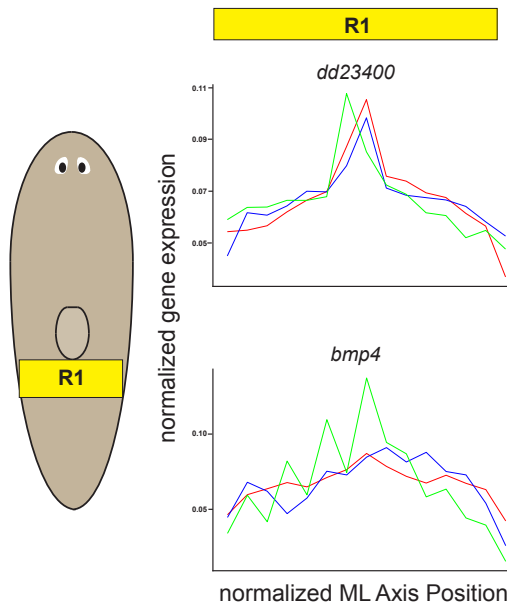**E**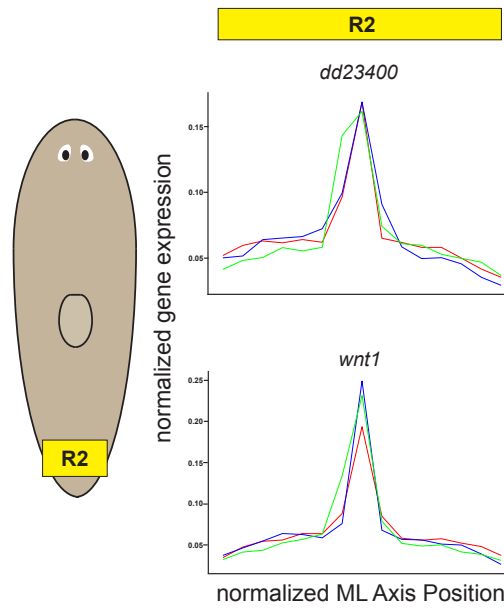**F**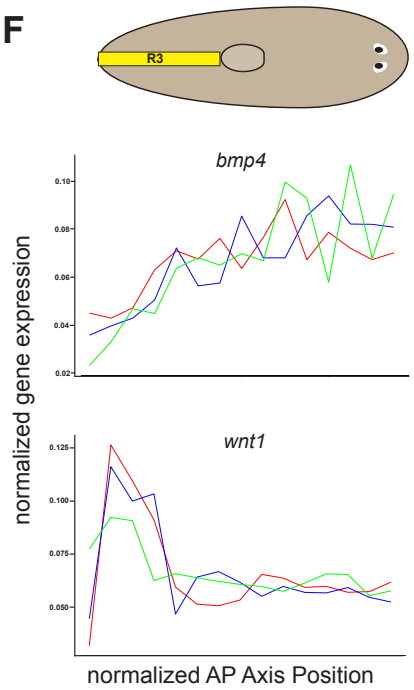

Supplement: S1 Fig — Analysis of normalized FISH line-scan intensity from dorsal-view images represented in Fig 1A measuring (A) mediolateral expression of bmp4 and dd23400 from an anterior tail domain, (B) mediolateral expression of dd23400 and wnt1 measured from a posterior tail domain, and (C) anteroposterior expression of wnt1 and bmp4 measured within the tail. 3 biological replicate images were processed in ImageJ to measure line-scan intensity with a width of 120 (A-B) or 30 pixels (C), encompassing approximately 1/10th the size of the orthogonal axis. Cartoons indicate the regions and approximate relative scale of measurements taken for each analysis (R1, R2, R3). Data were position-normalized and intensity-normalized, then summed within 15 equal-sized bins dividing each region. Averages and standard deviations across biological replicates for each bin are presented in each plot (A-C). Statistical tests were performed to assess overall expression trends using t-tests to compare sample intensities across the bins indicated by the dotted lines. t-test for ML-bmp4 distributions compares data from bins 1–3 versus bins 7–9 (N = 9 measurements for each region), t-tests for ML plots of dd23400 and wnt1 compares data from bins 4–6 versus bins 7–9 (N = 9 measurements for each region), and t-tests for AP bmp4 and wnt1 compares data from bins 1–4 versus bins 5–8 (N = 12 measurements for each region). (D-F) Plots of individual biological replicates after the normalization procedure described above. The key trends observed in this analysis are that bmp4 expression is present in a graded fashion on the mediolateral axis dorsally, dd23400 is expressed sharply at the midline, wnt1 is expressed at the midline in the posterior, and bmp4 expression along the posterior midline reduces in the far posterior at approximately the same location as wnt1 is expressed. (PDF) [file pgen.1010608.s001.pdf]

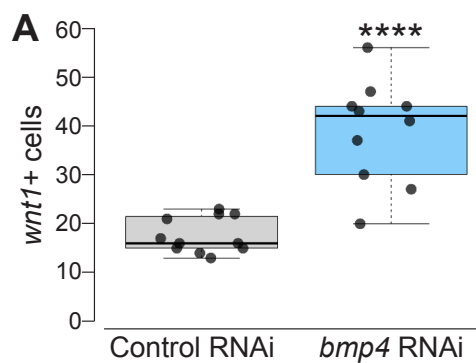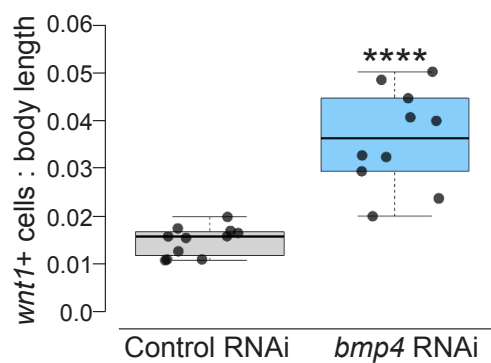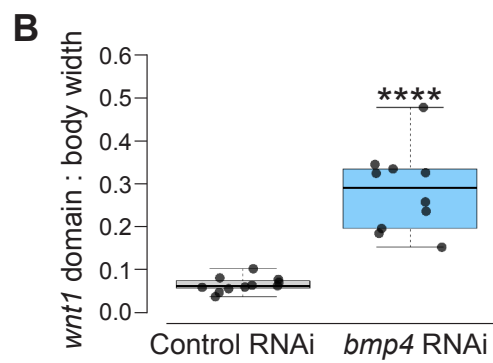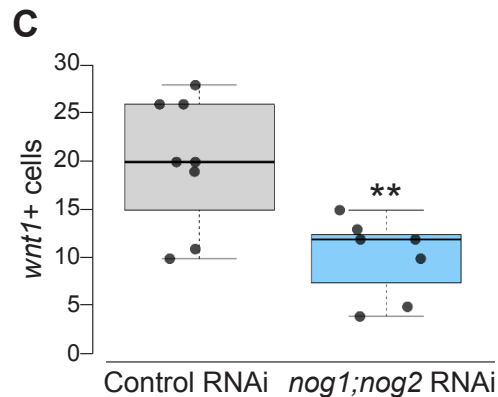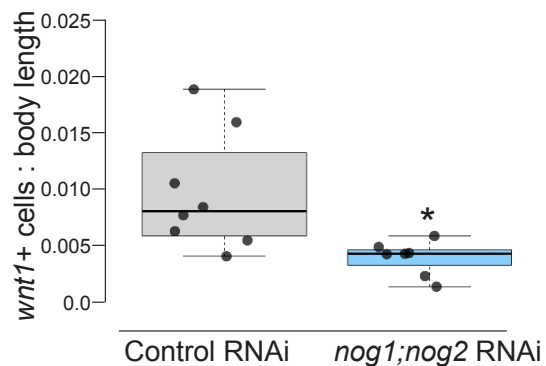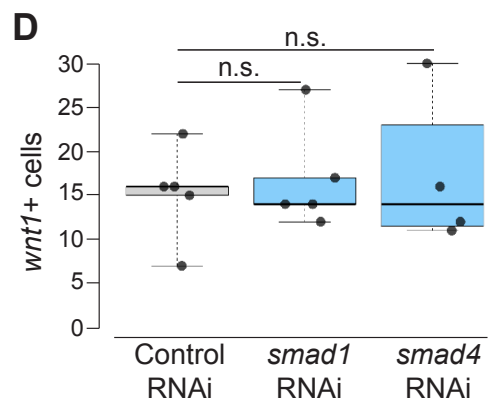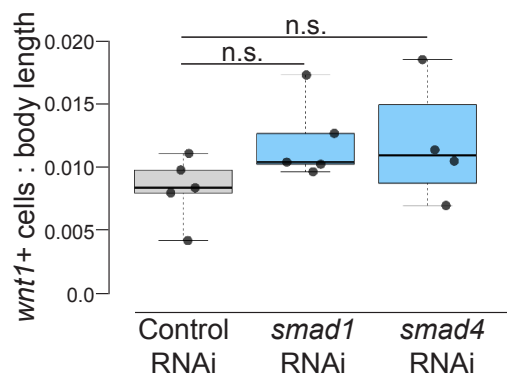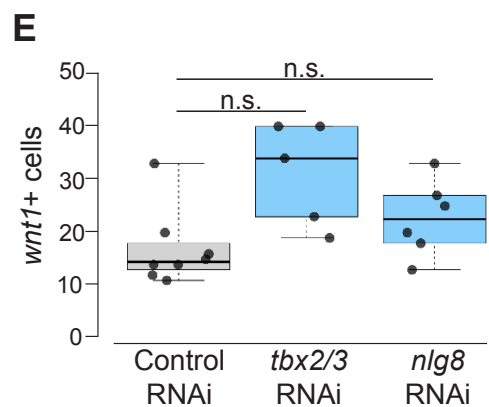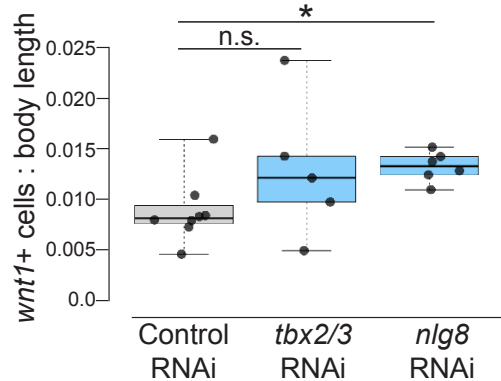

Supplement: S2 Fig — (A-E) Quantification of absolute and relative wnt1+ cell numbers after inhibition of indicated genes related to Fig 2. Graphs showing absolute numbers of wnt1+ cells and numbers of wnt1+ cells normalized to body length for (A) 14 days of control or bmp4 RNAi, (C) 18 days of control or nog1;nog2 RNAi, (D) 14 days of control, smad1, or smad4 RNAi, or (E) 18 days of control, tbx2/3, or nlg8 RNAi. (B) Graph illustrating the maximum width between wnt1+ cells relative to animal body width following 14 days of control or bmp4 RNAi. (A-E) N ≥ 4 animals. Plots shows median values (middle bars) and first-to-third interquartile ranges (boxes); whiskers indicate 1.5× the interquartile ranges and dots are data points from individual animals. *p<0.05, **p<0.01, ****p<0.0001, n.s. indicates p>0.05 by 2-tailed t-test (A-C) or by one-way ANOVA on ranks (D-E). (PDF) [file pgen.1010608.s002.pdf]

**A**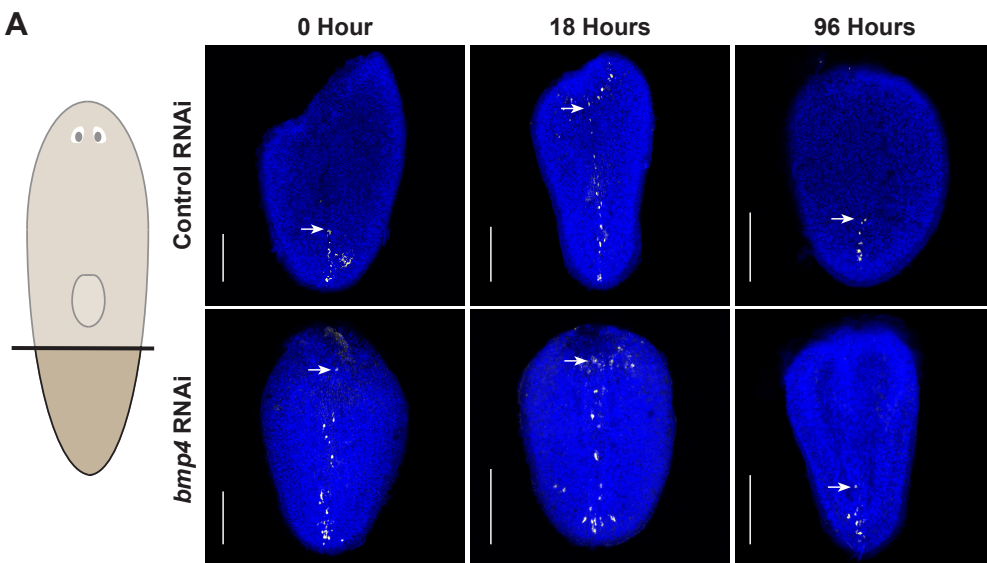**B**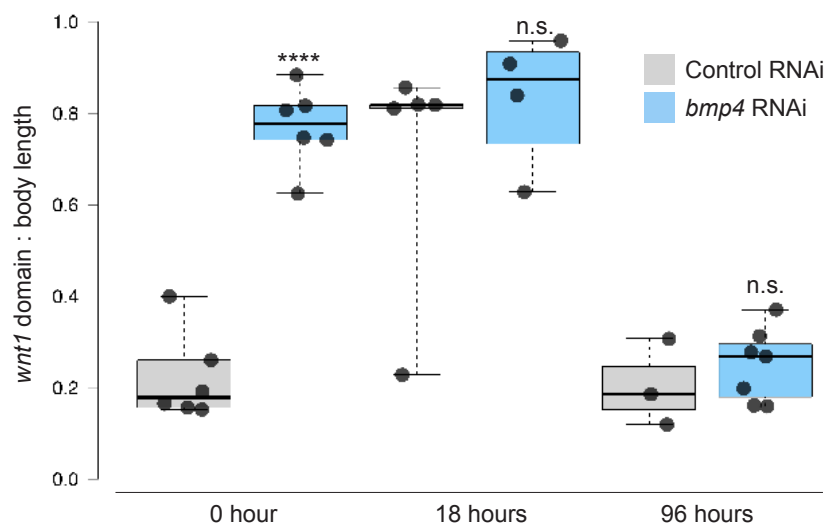

Supplement: S3 Fig — (A) FISH to detect wnt1 expression in regenerating tail fragments at 0, 18, and 96 hours after amputations conducted after 14 days of either control or bmp4 RNAi. Arrows indicate anterior-most wnt1+ cell detected along the dorsal midline for each timepoint and condition. Top panels show control animals undergoing early expansion of the dorsal midline wnt1 domain by 18 hours of regeneration followed by rescaling to reduce the domain to the tip of the animal by 96 hours. Bottom panel shows wnt1 expression dynamics in bmp4 RNAi, in which midline wnt1 expression was anterior expanded at the time of injury (0 hours), remained expanded at 18 hours of regeneration, and then restricted posteriorly by 96 hours, similar to control RNAi conditions. Therefore, BMP pathway modulation is unlikely to be responsible for the normal restriction of wnt1 by 96 hours in regenerating tail fragments. Scale bars represent 150 μm. (B) Graph showing the quantification of the length of the wnt1 domain relative to length of tail fragment. ****p<0.0001 by 2-tailed t-test and n.s. indicates p>0.05; N ≥ 3 animals. Box plots shows median values (middle bars) and first to third interquartile ranges (boxes); whiskers indicate 1.5× the interquartile ranges and dots are data points from individual animals. (PDF) [file pgen.1010608.s003.pdf]

Control RNAi   *bmp4* RNAi   Control RNAi   *bmp4* RNAi

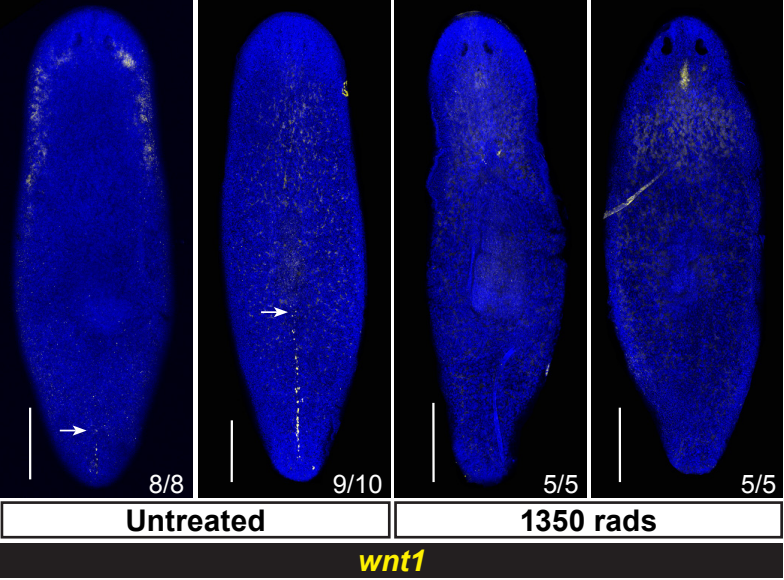

Supplement: S4 Fig — FISH detecting wnt1 in control and bmp4(RNAi) animals given 5 dsRNA dosings over 12 days following either no irradiation or given a sublethal dose of 1350 rads of X-ray irradiation. Unirradiated bmp4(RNAi) animals underwent expansion of wnt1 expression compared to unirradiated control RNAi conditions. By contrast, wnt1 expression was not present in irradiated control or bmp4(RNAi) animals. N ≥ 5. Scale bars represent 300 μm. (PDF) [file pgen.1010608.s004.pdf]

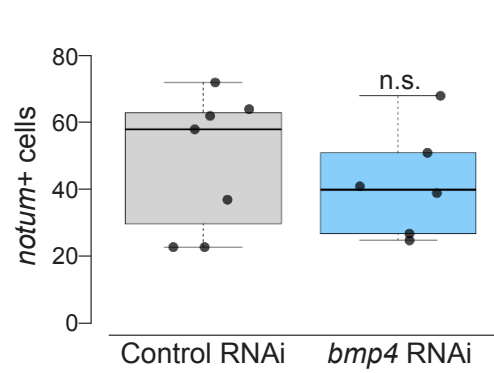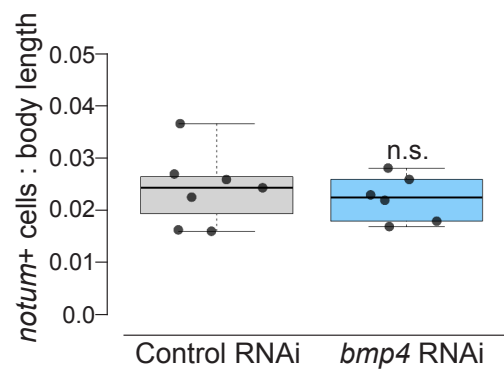

Supplement: S5 Fig — Plots of absolute and size-normalized numbers of anterior pole notum+ cells following homeostatic inhibition of bmp4 RNAi versus control RNAi animals, related to Fig 3B. Cells were manually scored from dorsal-view images obtained at 20x. Knockdown of bmp4 did not significantly change the absolute or bodysize-relative number of notum+ cells. n.s. indicates p>0.05 by 2-tailed t-test. N ≥ 6 animals. Plots shows median values (middle bars) and first-to-third interquartile ranges (boxes); whiskers indicate 1.5× the interquartile ranges and dots are data points from individual animals. (PDF) [file pgen.1010608.s005.pdf]

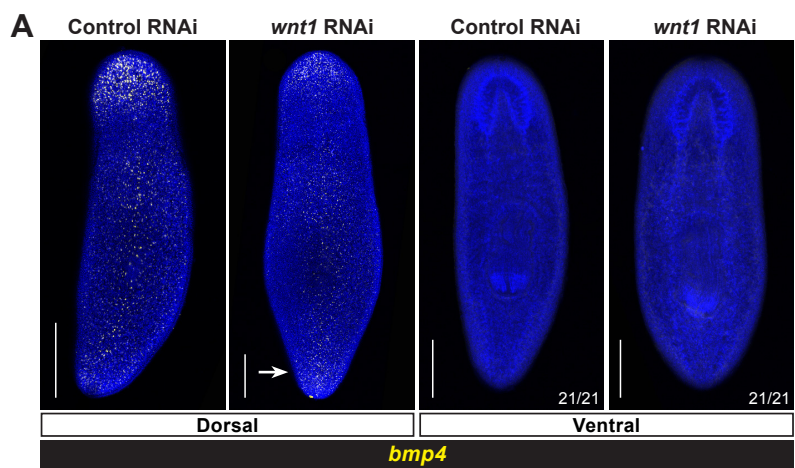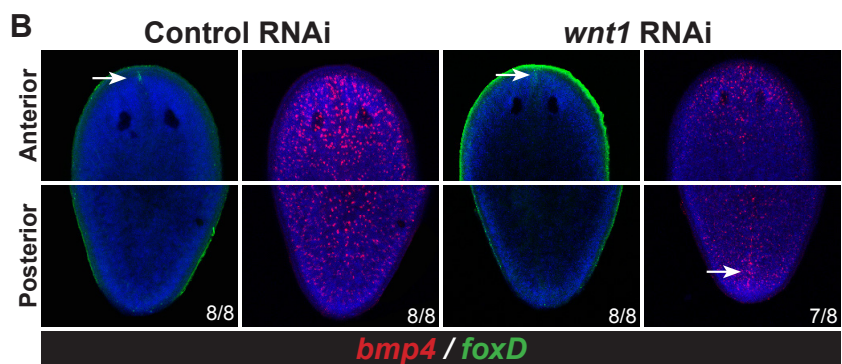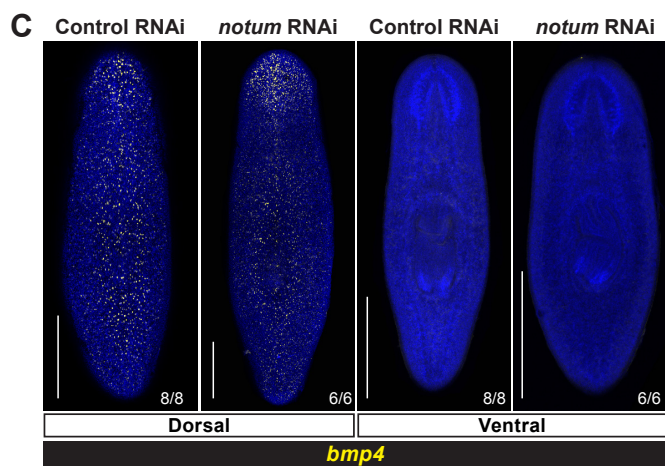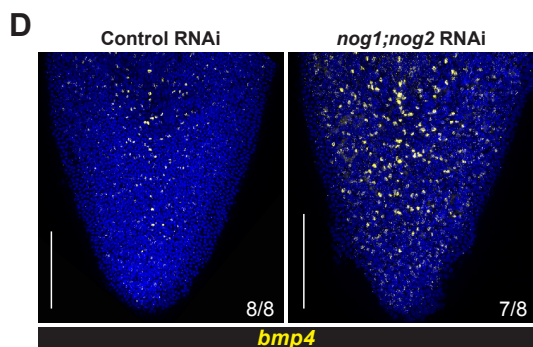

Supplement: S7 Fig — (A) FISH staining for bmp4 expression in whole animals after 14 days of control or wnt1 RNAi related to Fig 4. Inhibition of wnt1 resulted in elevated expression of bmp4 on the posterior midline of the animal (arrows) and did not change the lack of ventral bmp4 expression (right). Scale bars are 150 μm. N = 21 animals. (B) Double FISH of control or wnt1 RNAi for bmp4 and anterior marker foxD. N = 8. Inhibition of wnt1 caused elevated expression of bmp4 in the posterior but did not alter the lack of posterior foxD, suggesting wnt1’s role on bmp4 expression is not likely due to control of head-versus-tail identity determination. (C) Animals stained for bmp4 following control or notum RNAi homeostatically for 14 days. Inhibition of notum did not alter bmp4 expression on either the dorsal or ventral side of the animals. Scale bars are 300 μm. N ≥ 6. (D) Dorsal posterior view of bmp4 FISH conducted on control or nog1;nog2(RNAi) animals inhibited homeostatically for 18 days. nog1;nog2 inhibition qualitatively appeared to cause an increase in overall bmp4 expression levels. N = 8. Scale bars are 150 μm. (PDF) [file pgen.1010608.s007.pdf]

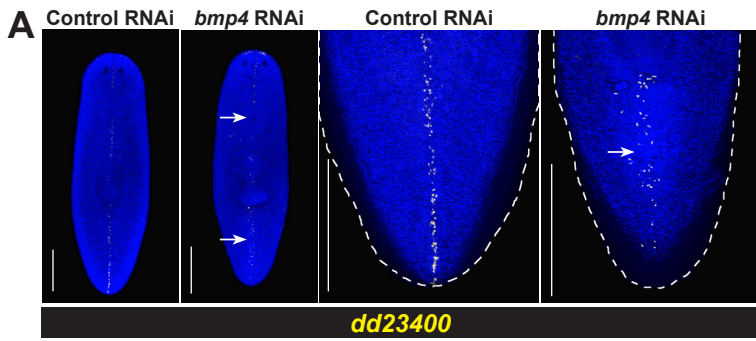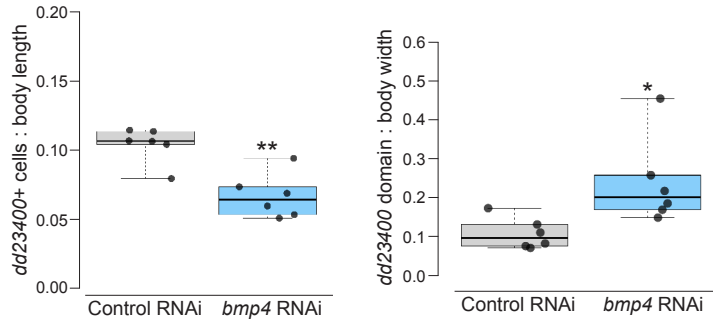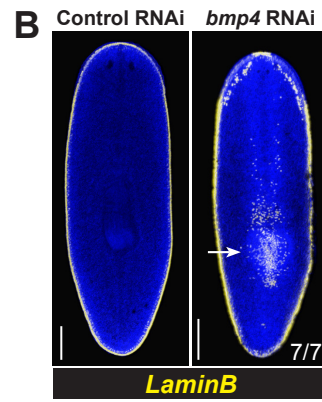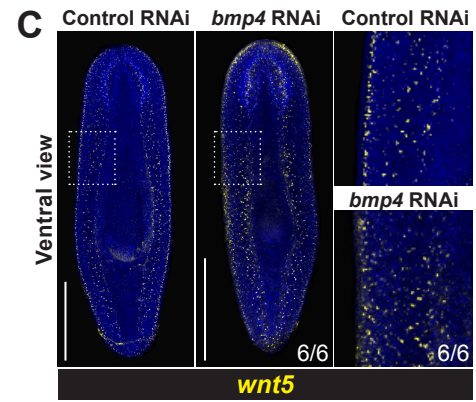

Supplement: S8 Fig — (A-C) FISH for dd23400, LaminB, and wnt5 following 28 days of control or bmp4 RNAi. Scale bars represent 300 μm. (A) Inhibition of bmp4 reduces dd23400 expression (arrows), particularly in the posterior. Bottom Left: Quantification of number of dd23400+ cells normalized to animal body length. Bottom Right: Quantification of maximum width between dd23400+ cells relative to animal body width. *p<0.05, **p<0.01 by unpaired 2-tailed t-test; N ≥ 6 animals. Box plots show median values (middle bars) and first to third interquartile ranges (boxes); whiskers indicate 1.5× the interquartile ranges and dots are data points from individual animals. (B) bmp4 RNAi causes ectopic medial expression of lateral marker laminB expression on the posterior midline (arrows). (C) Knockdown of bmp4 appears to elevate wnt5 expression less dramatically on the ventral side versus dorsal side. Right panels show enlargements of boxed regions. (PDF) [file pgen.1010608.s008.pdf]

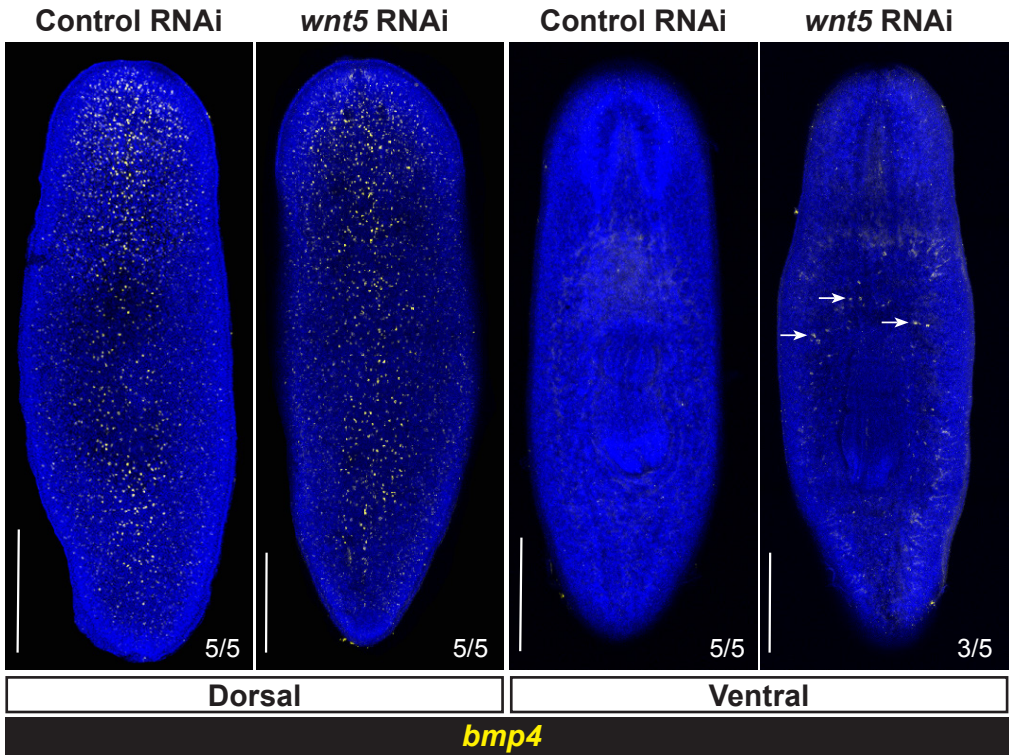

Supplement: S9 Fig — FISH staining for bmp4 after 21 days of control or wnt5 RNAi under homeostatic conditions. Inhibition of wnt5 does not cause detectable increases or decreases in bmp4 expression or distribution on the dorsal side of animals. However, some ectopic ventral expression appears following wnt5 RNAi. Scale bars are 150 μm. N = 5 animals. (PDF) [file pgen.1010608.s009.pdf]

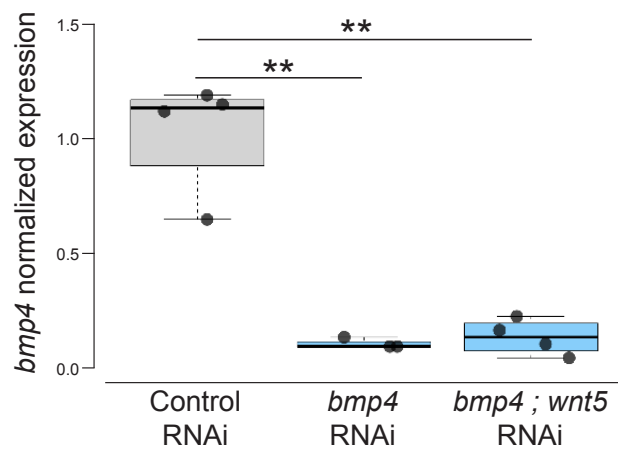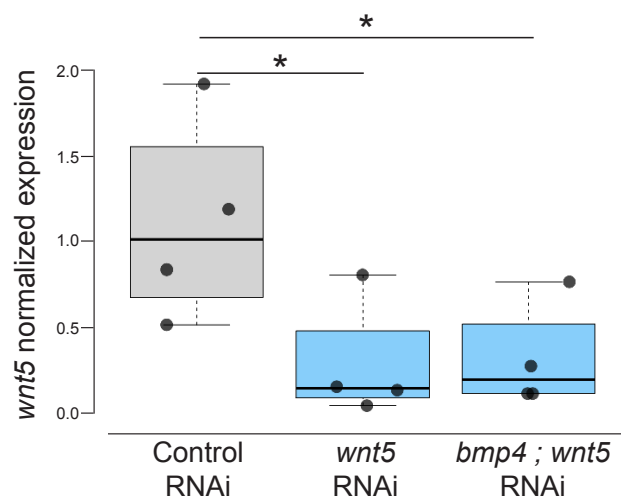

Supplement: S10 Fig — Left: qPCR to detect expression of bmp4 transcript (normalized to ubiquilin control) to detect knockdown of bmp4 in bmp4 RNAi and bmp4;wnt5 RNAi. Animals were treated with dsRNA for 20 days (9 dsRNA feedings), followed by isolation of RNA, followed by RT-qPCR. bmp4 expression was knocked down after delivery of either bmp4 dsRNA or the combination of bmp4 and wnt5 dsRNA. Right: qPCR to detect expression of wnt5 after RNAi of wnt5 individually or in combination with bmp4 under the same conditions. Both single and double RNAi conditions caused significant wnt5 knockdown. *p<0.05, **p<0.01 by one-tailed t-test to determine if mRNA reduced after RNAi. Plots show median values (middle bars) and first-to-third interquartile ranges (boxes); whiskers indicate 1.5× the interquartile ranges and dots are data points from individual animals. (PDF) [file pgen.1010608.s010.pdf]

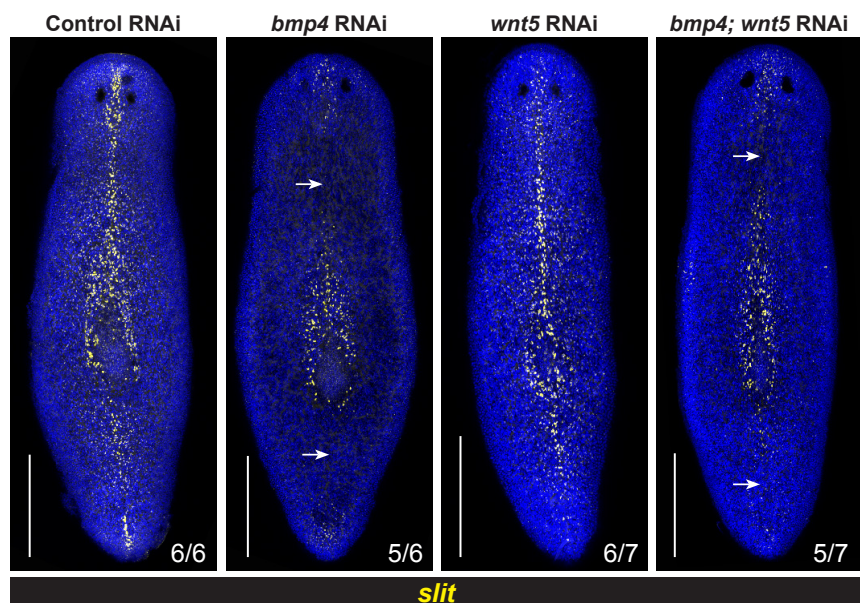

Supplement: S11 Fig — FISH staining for slit following 20 days of control, bmp4, wnt5, and bmp4;wnt5 RNAi. Loss of bmp4 reduces slit expression (arrows). Loss of wnt5 did not appear to alter slit expression. Knockdown of both bmp4 and wnt5 resulted in the reduced slit expression, similar to bmp4 RNAi (arrows). N ≥ 6 animals. Scale bars represent 300 μm. (PDF) [file pgen.1010608.s011.pdf]

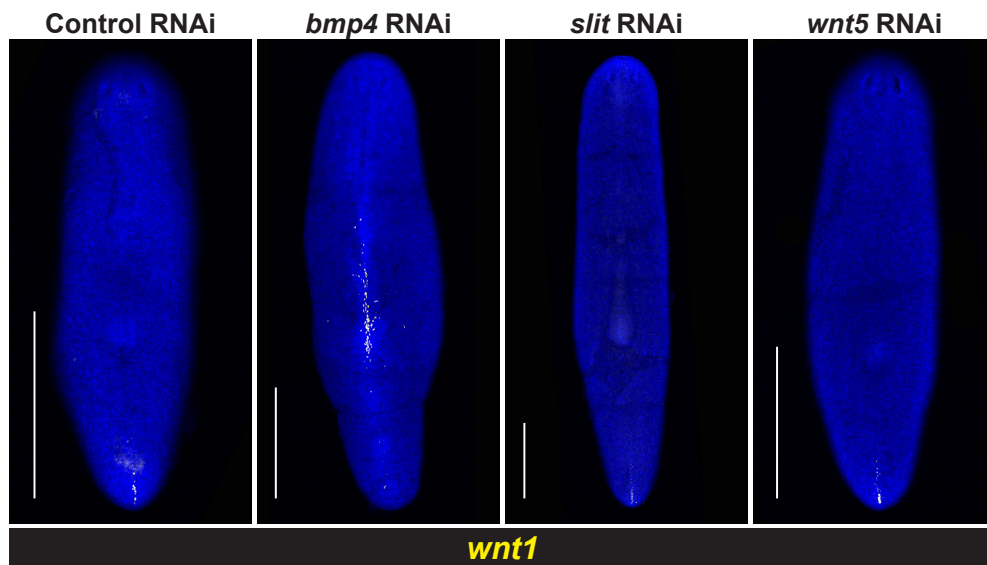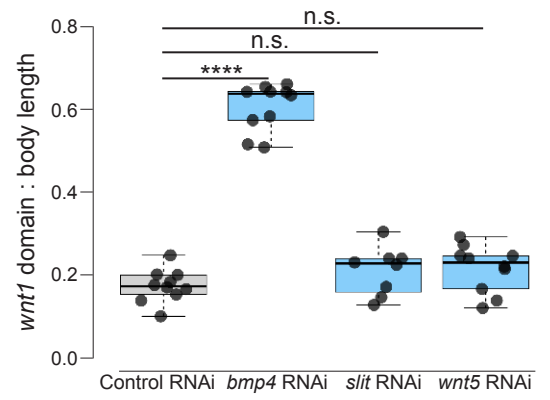

Supplement: S12 Fig — Left: Following 18 days of control, bmp4, slit, or wnt5 RNAi, animals were stained for wnt1 expression. Inhibition of bmp4 expanded wnt1 anteriorly and reduced posterior expression. Inhibition of slit or wnt5 did not significantly affect wnt1 expression. N ≥ 6 animals. Scale bars represent 300 μm. Right: Plot showing length of wnt1 domain from the tip of the tail relative to animal body length. n.s. indicates p>0.05, ****p<0.0001 by one-way ANOVA on ranks. N ≥ 8 animals. Plots show median values (middle bars) and first-to-third interquartile ranges (boxes); whiskers indicate 1.5× the interquartile ranges and dots are data points from individual animals. (PDF) [file pgen.1010608.s012.pdf]
