# Supplementary material for: BMP suppresses WNT to integrate patterning of orthogonal body axes in adult planarians
Source: PLoS Genet. 2023 Sep 20;19(9):e1010608. doi: 10.1371/journal.pgen.1010608 (PMC10545109; doi:10.1371/journal.pgen.1010608)
Supplement: S1 Table — (PDF) [file pgen.1010608.s013.pdf]

Table S1. Primer sequences

Gene name	ddv6	Left Primer	Right Primer	Primer Use
bmp4	dd_Smed_v6_17402_0_1	TCCATCAGAGAAAGTTCGCAGT	ACGAATGTACAGTTTCAGTTGCA	riboprobe, dsRNA
dd23400	dd_Smed_v6_23400_0_1	GCCCATCAGATGTCCGCTTA	TTTTCCAACACAGTGCAACACA	riboprobe, dsRNA
wnt1	dd_Smed_v6_28398_0_1	CCTCAAAATCGAATTTTACACTCA	TGGGACAAAAATAAAATTCCACA	riboprobe, dsRNA
nog1	dd_Smed_v6_764_0_1	CAAATGAAAGATTTCGAGGTGA	TGTTGAACAGAGCATCAATGAA	dsRNA
nog2	dd_Smed_v6_1134_0_1	CCACATCGACATGTTAGCAA	ATCGCGACAATGTCTCACAG	dsRNA
smad1	dd_Smed_v6_6877_0_1	CATCTCCGGCTGTTAAACGG	CCACAAGAATAAAGCAAAATGGCA	dsRNA
smad4	dd_Smed_v6_1923_0_1	CTTCAAATTGCCGGGCGAAA	TCGAGTATCGCGCTGATTCC	dsRNA
tbx2/3	dd_Smed_v6_11693_0_1	GCTTCGTTTCTGCCGAGTTT	CGGATGGGTTTTGAATCGCG	dsRNA
nlg8	dd_Smed_v6_8738_0_1	CCTCCACGTGAATCCACAGT	TCGGTTTCCAGAACTGAGAAA	dsRNA
notum	dd_Smed_v6_24180_0_1	AAAATTTCTGAGGATCGAAAAA	TGAAGCTAGATTTATGTGAAAAACCA	riboprobe, dsRNA
sfrp-1	dd_Smed_v6_13985_0_1	TTGAATTCATGGAAATGACCAA	AATCAATGAAATGTTTTGTTGTGA	riboprobe
ndl-5	dd_Smed_v6_5102_0_1	ACAGTATTTCTTAACACGGGTCA	TGAACCATACGGAGCGGT	riboprobe
wntP-2	dd_Smed_v6_7326_0_1	TTAAATGTTCTAAGCCAAAACAACA	AAAACTTTTATGATCAATCTGAATGC	riboprobe
fzd4-1	dd_Smed_v6_11650_0_1	GGAATAGCCCAACTCACCAA	TGCCGAATTTAGTTGGAAGC	riboprobe
wnt11-1	dd_Smed_v6_14391_0_1	CATGAGCCAGTAAATGAAATGGT	TAGCACTGCGTTGGTGTTTG	riboprobe
wn11-2	dd_Smed_v6_16209_0_1	TTGATCGCATGAAAAATTACAAA	CCATTGCAATAAAATTGTCCA	riboprobe
netrin-2	dd_Smed_v6_14852_0_1	CAGACCGACACATTCCAAAA	GTTCACATTTTCCGCGTTTT	riboprobe
kal-1	dd_Smed_v6_6746_0_1	CGAGTTCTGAACCTGGCTGT	GTTTCCCCTCCTGTGTTGAA	riboprobe, dsRNA
slit	dd_Smed_v6_12111_0_1	TCCGTGACAATCAGCTGCAA	AAGCCGATGATTGCCGAGAA	riboprobe, dsRNA
wnt5	dd_Smed_v6_15469_0_1	TCGGGGTGACCTTTTACTCA	TGAACCCATTGCAGTGAAAA	riboprobe, dsRNA
SMU15007112	dd_Smed_v6_9120_1_1	CCCCGTGTGGATATTTCAGT	AGCAAAATCGGTTCTCCGTA	riboprobe
foxA	dd_Smed_v6_10718_0_1	AACGACCTCAACGGAATGTTT	CATGCGCCAAAGTTAAGGATA	riboprobe
laminin	dd_Smed_v6_8356_0_1	AGTCGCTGGCAAAGTGCATCT	AATGATGCGTGGTATCCACAG	riboprobe
laminB	dd_Smed_v6_3065_0_1	AGTTCAACTCCTGGCGCTAC	CTGAACATTCCGGATCACCT	riboprobe
ubiquilin	dd_Smed_v6_1364_0_1	AAATTCGCCTGCCTGTTGGG	CCGGTGGCATTAATCCATCTGT	qRT-PCR
bmp4	dd_Smed_v6_17402_0_1	TGGATCATAGCCCCTCAGAA	TGCATGGTTTGTGGCATTGA	qRT-PCR
wnt5	dd_Smed_v6_15469_0_1	GATCACATGCCAGCAGTCAG	TCAGCTGCCTTTGACACTCT	qRT-PCR
