# Supplementary material for: BMP suppresses WNT to integrate patterning of orthogonal body axes in adult planarians
Source: PLoS Genet. 2023 Sep 20;19(9):e1010608. doi: 10.1371/journal.pgen.1010608 (PMC10545109; doi:10.1371/journal.pgen.1010608)
Supplement: S6 Fig — (A) Following 28 days RNAi of control or bmp4 RNAi, animals were stained for gut marker porcupine and pharynx-associated markers SMU15007112, dd554, foxA, and laminin. Inhibition of bmp4 did not affect porcupine or SMU15007112 expression. bmp4 RNAi caused a subtle change to dd554 expression which kept overall pharynx staining intact but eliminated expression of streams of dd554+ cells located outside of the pharynx (zoom-ins, 6/7 animals had no dd554 expressing cells adjacent to the pharynx, compared with 7/7 control animals displaying this expression). However, inhibition of bmp4 consistently resulted in ectopic non-pharynx expression of foxA and ectopic anterior expression of laminin. 5/8 bmp4(RNAi) animals had relatively less reduction to laminin pharynx staining and less ectopic anterior laminin and 3/8 animals had coincident loss of laminin expression and stronger gain of ectopic anterior expression. N ≥ 7. Scale bars represent 300 μm. (B) Measurements of head, pharynx, and tail regions determined by dd554 expression relative to body region for control and bmp4 RNAi. bmp4 inhibition did not significantly alter AP pharynx scale or position or size of tail and anterior (head) domain as measured with respect to pharynx position. (C) Left: Hoechst-stained animals following 28 days of control or bmp4 RNAi to detect original and ectopic eyes. Right: Quantification of the length from the anterior tip of the eyes (as measured for each pair of eyes along the midline) and normalized to total body size. Top panels show zoom-in of anterior animal regions for control and bmp4(RNAi) animals of eyes. Images are max-projections with z-planes chosen to show only either the original or ectopic bmp4(RNAi) animal eyes as indicated. Compared to the position of control eyes, original eyes in bmp4(RNAi) animals were displaced anteriorly toward the tip of the head while ectopic eyes are positioned at the same relative location as normal eyes in control animals. Scale bars represent 30 [file pgen.1010608.s006.pdf]

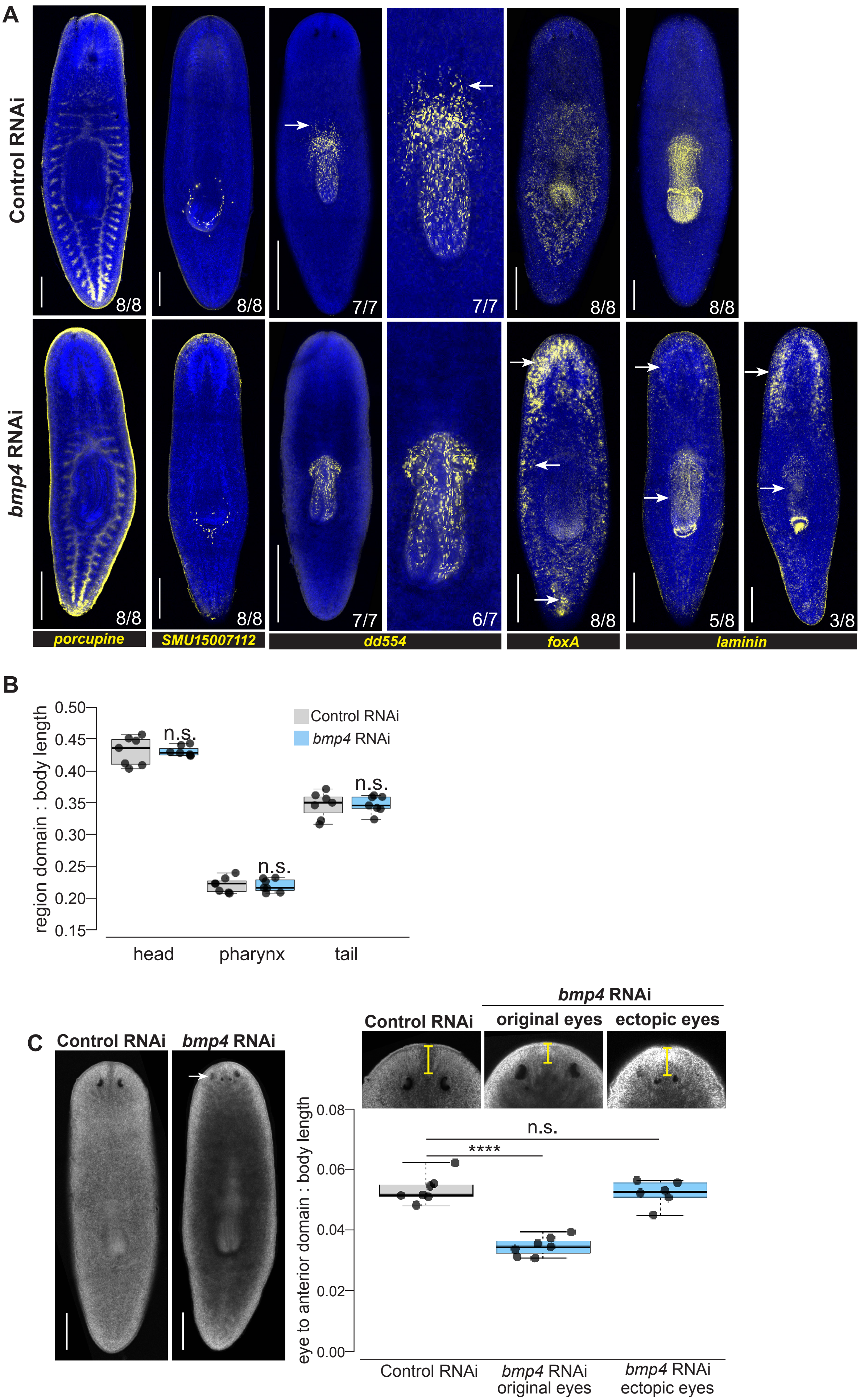
A
Control RNAi
bmp4 RNAi
8/8
8/8
7/7
7/7
8/8
8/8
8/8
8/8
7/7
6/7
8/8
5/8
3/8
porcupine
SMU15007112
dd554
foxA
laminin
B
region domain : body length
0.50
0.45
0.40
0.35
0.30
0.25
0.20
0.15
n.s.
n.s.
n.s.
Control RNAi
bmp4 RNAi
head
pharynx
tail
C
Control RNAi
bmp4 RNAi
bmp4 RNAi
Control RNAi
original eyes
ectopic eyes
eye to anterior domain : body length
0.08
0.06
0.04
0.02
0.00
n.s.
****
Control RNAi
bmp4 RNAi original eyes
bmp4 RNAi ectopic eyes
